# Quantifying spatial heterogeneity of chlorophyll fluorescence during plant growth and in response to water stress

**DOI:** 10.1186/s13007-015-0067-5

**Published:** 2015-03-26

**Authors:** Justine Bresson, François Vasseur, Myriam Dauzat, Garance Koch, Christine Granier, Denis Vile

**Affiliations:** Laboratoire d’Ecophysiologie des Plantes sous Stress Environnementaux (LEPSE), INRA, Montpellier SupAgro, UMR759, F-34060 Montpellier, France; Laboratoire des Symbioses Tropicales et Méditerranéennes (LSTM), UMR113, Université Montpellier 2-IRD-CIRAD-INRA-SupAgro, F-34095 Montpellier, France; Center for Plant Molecular Biology (ZMBP), General Genetics, University of Tuebingen, D-72076 Tuebingen, Germany; Max Planck Institute for Developmental Biology, D-72076 Tuebingen, Germany

**Keywords:** *Arabidopsis thaliana*, Chlorophyll fluorescence imaging, Heterogeneity of *F*_v_/*F*_m_ values, Modelling, Photosynthetic performance, Pixels distribution, Plant growth, Plant survival, Sensitivity analysis

## Abstract

**Background:**

Effects of abiotic and biotic stresses on plant photosynthetic performance lead to fitness and yield decrease. The maximum quantum efficiency of photosystem II (*F*_v_/*F*_m_) is a parameter of chlorophyll fluorescence (ChlF) classically used to track changes in photosynthetic performance. Despite recent technical and methodological advances in ChlF imaging, the spatio-temporal heterogeneity of *F*_v_/*F*_m_ still awaits for standardized and accurate quantification.

**Results:**

We developed a method to quantify the dynamics of spatial heterogeneity of photosynthetic efficiency through the distribution-based analysis of *F*_v_/*F*_m_ values. The method was applied to *Arabidopsis thaliana* grown under well-watered and severe water deficit (survival rate of 40%). First, whole-plant *F*_v_/*F*_m_ shifted from unimodal to bimodal distributions during plant development despite a constant mean *F*_v_/*F*_m_ under well-watered conditions. The establishment of a bimodal distribution of *F*_v_/*F*_m_ reflects the occurrence of two types of leaf regions with contrasted photosynthetic efficiency. The distance between the two modes (called S) quantified the whole-plant photosynthetic heterogeneity. The weighted contribution of the most efficient/healthiest leaf regions to whole-plant performance (called *W*_max_) quantified the spatial efficiency of a photosynthetically heterogeneous plant. Plant survival to water deficit was associated to high S values, as well as with strong and fast recovery of *W*_max_ following soil rewatering. Hence, during stress surviving plants had higher, but more efficient photosynthetic heterogeneity compared to perishing plants. Importantly, S allowed the discrimination between surviving and perishing plants four days earlier than the mean *F*_v_/*F*_m_. A sensitivity analysis from simulated dynamics of *F*_v_/*F*_m_ showed that parameters indicative of plant tolerance and/or stress intensity caused identifiable changes in S and *W*_max_. Finally, an independent comparison of six Arabidopsis accessions grown under well-watered conditions indicated that S and *W*_max_ are related to the genetic variability of growth.

**Conclusions:**

The distribution-based analysis of ChlF provides an efficient tool for quantifying photosynthetic heterogeneity and performance. S and *W*_max_ are good indicators to estimate plant survival under water stress. Our results suggest that the dynamics of photosynthetic heterogeneity are key components of plant growth and tolerance to stress.

**Electronic supplementary material:**

The online version of this article (doi:10.1186/s13007-015-0067-5) contains supplementary material, which is available to authorized users.

## Background

High-throughput phenotyping is increasingly used for dissecting the genetic and eco-physiological determinisms of plant performance and stress tolerance. Over the last decade, efficient automated imaging systems have been developed for the acquisition of visible, bioluminescence, fluorescence and multi-spectral images. A rising difficulty is now to extract valuable, *i.e.*, biologically meaningful, preferably quantitative, information from the large collection of images generated by these systems [1].

Chlorophyll fluorescence (ChlF) imaging has become one of the most powerful and popular tools to track changes in the photosynthetic capacities of plants in response to abiotic and biotic factors [2-4]. Pulse-amplitude modulated ChlF techniques provide non-invasive assessment of the photosystem II (PSII) efficiency to supply electrons to the photosynthetic machinery. Light energy absorbed by chlorophyll molecules can undergo one of three competing fates: (*i*) driving photosynthesis (photochemistry); (*ii*) being dissipated as heat; or (*iii*) being re-emitted as ChlF. These three processes take place in a competitive manner, and under stress conditions, the photochemistry declines whereas heat dissipation and ChlF emission increase (for recent reviews, see [5,6]). ChlF is estimated by the quantification of the light re-emitted (in the red wavebands) after the application of a saturating flash (usually for a few seconds) to the photosynthetic organs [5]. The saturating flash induces the transport of electrons through PSII centres, driving the reduction of Q_A_, the primary stable electron acceptor of PSII. Once reduced, Q_A_ cannot accept new electrons before electrons are transferred to the next acceptor (the reaction centre is considered to be ‘closed’), and the excess of energy is dissipated through heat and fluorescence.

Amongst the different ChlF parameters, the *F*_v_/*F*_m_ ratio is a useful and rapid parameter that reflects the maximum quantum efficiency of the PSII photochemistry [7]. In dark-adapted leaves (in which all PSII reaction centres are in the ‘open’ state; Q_A_ fully oxidized), a measuring beam is applied to elicit the minimal value of ChlF, *F*_0_ (*i.e.,* basal fluorescence). *F*_0_ represents the energy dissipation via light-harvesting antenna pigments when excitation energy is not being transferred to the PSII reaction centres. After reaching *F*_0_, the application of a brief saturating pulse induces a maximum value of ChlF, *F*_m_ (PSII reaction centres get ‘closed’ because of electron accumulation; Q_A_ fully reduced). The difference between *F*_0_ and *F*_m_ is the variable fluorescence, *F*_v_ and *F*_v_/*F*_m_ is given by (*F*_m_-*F*_0_)/*F*_m_ (for more details, see [5]). Low *F*_v_/*F*_m_ indicate substantial photoinhibition or down-regulation of PSII that occurs when plants experience stress. It has been shown that *F*_v_/*F*_m_ is a robust indicator of plant health. Healthy photosynthetic tissues of most plant species exhibit a mean *F*_v_/*F*_m_ at *ca.* 0.83, while lower values are indicative of an impaired physiological status [8,9]. Rapid modifications of *F*_v_/*F*_m_ are for instance reported in response to many environmental factors, such as water stress [8,10], temperature [11-13], wounding [14], photoinhibition [11,15], biotic interactions such as pathogenic as well as beneficial bacteria [16-19].

Soil water availability is one of the most important environmental factors for plant growth and development. The impact of water deficit on the photosynthetic performance of plants depends on the severity and duration of the stress. In the short-term, decrease in water supply usually induces stomata closure to maintain a favourable leaf water status, what in turn leads to a reduction of internal CO_2_ concentration [20]. Hence, stomata closure under water stress promotes an imbalance between the photochemical activity of PSII and the electron requirement for carbon fixation, leading to over-excitations and subsequent photoinhibitory damages to PSII reaction centres [21]. As a consequence, substantial decline in *F*_v_/*F*_m_ in response to moderate water deficit is observed in various plant species (see references in [2]), and was closely related to decreased relative leaf water content [8]. With increasing stress severity or duration, carbon starvation and hydraulic failure, which strongly alter *F*_v_/*F*_m_ at the whole-plant level, lead to partial (or total) senescence or leaf abscission [22]. Even though exacerbated leaf senescence can be lethal, sacrificing a few leaves might be a good strategy to ensure survival under severe resource limitation [23]. Growth recovery following severe water stress is then associated with the (partial) re-establishment of the photosynthetic capacities of the senescing leaves, and/or with the development of new leaves with optimal photosynthetic performance [24].

ChlF imaging has revealed that photosynthetic performance is extremely heterogeneous at the leaf surface, as well as between leaves, when plants experience environmental stresses. For examples heterogeneity in ChlF is reported in response to changing CO_2_ concentration [25], light stimuli [26], ozone-induced perturbations [27], low growth temperature [28], chilling [29], pathogen attack [16], drought [10,30] or treatment with abscisic acid [31]. Spatio-temporal heterogeneity across photosynthetic areas has been assessed by visual inspection of leaves [24,26,30], by measurements at spatially different small areas on the leaf surface [10,29,30], or by visual inspection of the shape of *F*_v_/*F*_m_ distributions across leaves [26,28,29,32]. ChlF imaging of leaves of Arabidopsis grown under water stress for instance reveals a progressive decline of *F*_v_/*F*_m_ beginning at the leaf’s tip [10]. However we still lack an automatic and standardized method for the quantification of the spatial heterogeneity of *F*_v_/*F*_m_ values, which is crucial to compare photosynthetic performance depending on the developmental stage, the genotype, or the environmental conditions.

Here, we analysed the distribution of *F*_v_/*F*_m_ to estimate the spatial heterogeneity of photosynthetic efficiency (S) and the weighted contribution of the most efficient/healthiest leaf regions to whole-plant photosynthetic performance (*W*_max_). We first showed that the changes in S and *W*_max_ were related to the survival of the Arabidopsis Col-0 accession to a severe water deficit (SWD). A sensitivity analysis of S and *W*_max_ to simulated dynamics of *F*_v_/*F*_m_ distributions showed to what extent S and *W*_max_ can vary depending on plant tolerance and/or stress intensity. Finally, we found that a significant part of the variation in biomass accumulation in six contrasted Arabidopsis accessions is explained by the variation of *W*_max_ in the course of plant development.

## Results

### Analytical framework: severe water deficit strongly affects plant growth, photosynthetic efficiency and induces plant mortality

**Figure 1:**
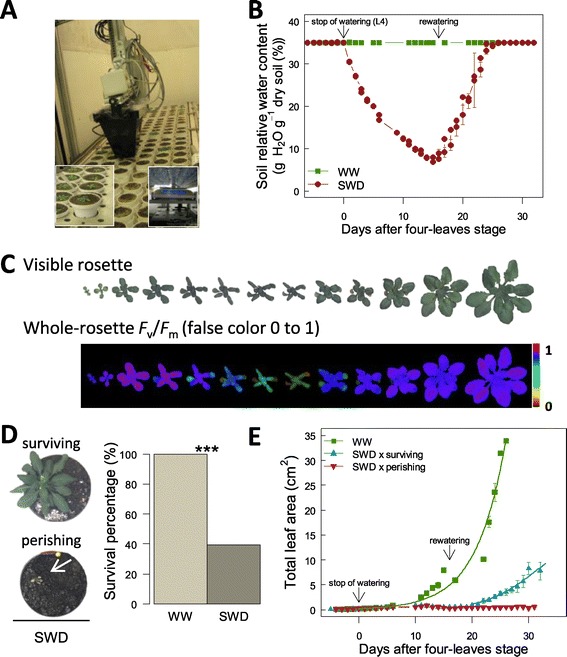
**High-throughput analysis of Arabidopsis growth and chlorophyll fluorescence in the PHENOPSIS automaton. (A)** *A. thaliana* plants are grown in controlled environmental conditions in the PHENOPSIS platform equipped with a chlorophyll fluorescence imaging system. **(B)** Dynamics of soil relative water content in two watering scenarios including constant well-watered conditions (WW) and water withdrawing from the four-leaves stage (L4; beginning of stress) followed by rewatering after 1 day at 6% g H_2_O^−1^ dry soil (SWD). Data are means (± SE) of 13 and 48 plants under WW and SWD, respectively. **(C)** Plant growth (top) and whole-rosette *F*_v_/*F*_m_ (bottom) during plant development and under SWD. *F*_v_/*F*_m_ values are represented by false colour scale ranging from black (pixel values 0) through red, yellow, green, blue to purple (ending at 1). **(D)** Visible images of surviving and perishing plants (left) and survival percentage of plants under WW and SWD conditions (right). Asterisks indicate significant differences following Chi^2^ test between plants grown in WW conditions *(n* = 13) and plants under SWD (*n* = 19 and 29 for surviving and perishing plants, respectively; ***: *P* < 0.001). **(E)** Total projected leaf area of plants under WW conditions and SWD (surviving and perishing plants) as a function of days after L4 stage until bolting. Data are means (± SE) of 13–29 plants.

Arabidopsis Col-0 plants were grown in the PHENOPSIS automaton [33] (Figure 1A). Plants were subjected to SWD by withholding irrigation from the four-leaves stage (L4; stage 1.04, [34]; Figure 1B) in order to progressively reach a very low soil relative water content (RWC_soil_) of 6% g H_2_O g^−1^ dry soil (corresponding to water potential *ca*. of −9.52 MPa; see Additional file 1: Figure S1). Thereafter, irrigation was resumed to progressively reach the well-watered (WW) soil condition (35% g H_2_O g^−1^ dry soil; 0.07 Mpa, Additional file 1: Figure S1) maintained until the flowering of surviving plants (Figure 1B). These two soil conditions allowed the investigation of *F*_v_/*F*_m_ heterogeneity with highly contrasted physiological status and thus, with a wide range of leaf damages and senescence. Plant growth and *F*_v_/*F*_m_ were daily measured from early developmental stages (*i.e.*, emergence of the two first leaves, stage 1.02, [34]) to the emergence of the flowering stem (*i.e.*, bolting, stage 5.01, [34]; Figure 1C), with a high-throughput ChlF imaging system (Imaging-PAM M-Series, Maxi-version, Heinz Walz GmbH, Germany) implemented on the automaton (Figures 1A, C). We developed an ImageJ (1.47v, Rasband, Bethesda, Maryland, USA) macro “PHENOPSIS-Fluo” to semi-automatically extract the whole-rosette *F*_v_/*F*_m_ mean, the distribution of *F*_v_/*F*_m_ values across the rosette and the projected total leaf area from ChlF images.

**Figure 2:**
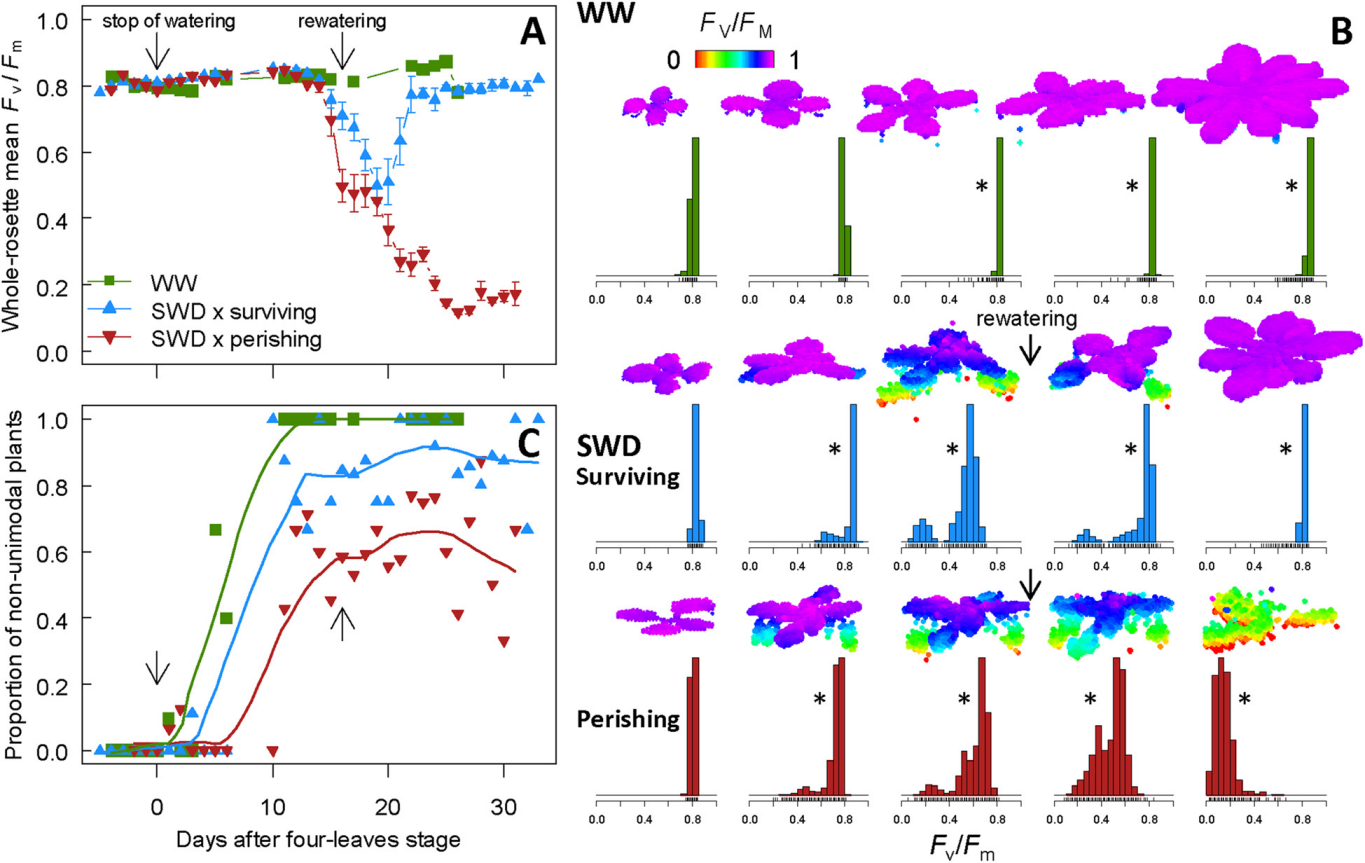
**Effects of severe water deficit on plant photosynthetic efficiency. (A)** Dynamics of whole-rosette mean *F*_v_/*F*_m_ of well-watered (WW) plants and stressed (surviving and perishing) plants under severe water deficit (SWD) as a function of days after four-leaves stage (L4; beginning of SWD) until bolting. Data are means (± SE) of 13–29 plants. **(B)** 3-D representations of vegetative rosettes under WW and SWD conditions in *F*_v_/*F*_m_ false colour (from black pixel values (0) through red, yellow, green, blue to purple (ending at 1)) and their corresponding *F*_v_/*F*_m_ distributions during time courses. Asterisks indicate *p*-value < 0.01 (Hartigan’s dip test) meaning significant departure from unimodality of *F*_v_/*F*_m_ values. Arrows indicate rewatering step. **(C)** Dynamics of the proportion of non-unimodal (*i.e.*, multimodal) plants under WW and SWD after L4 stage until bolting following the Hartigan’s dip test.

Under SWD, 40% of the plants survived, resumed growth and flowered whereas the remaining plants failed to recover, perished and decomposition of tissues started (Figures 1D, E). Whole-rosette mean *F*_v_/*F*_m_ followed the variation of RWC_soil_ and was therefore dramatically affected by the SWD (Figures 1B and 2A). Whole-rosette mean *F*_v_/*F*_m_ of stressed plants remained stable at 0.812 ± 0.041 (n = 4–30) during the 14 days after water withholding, similar to plants grown under WW conditions (0.813 ± 0.019; n = 4–31; Figure 2A). Then, whole-rosette mean *F*_v_/*F*_m_ of stressed plants decreased dramatically (Figure 2A). This was mainly due to the decrease of *F*_v_/*F*_m_ in the oldest leaves of the rosette, notably with a gradient from the tip to the base of the leaves (see 3-D representations in Figure 2B and Additional file 2: Figure S2). Just before rewatering, SWD resulted in a significant 38% and 43% decrease of mean *F*_v_/*F*_m_ in surviving and perishing plants, respectively (Figure 2A). Upon rewatering, mean *F*_v_/*F*_m_ continued to decline steadily for three further days. Afterwards, surviving plants progressively recovered *F*_v_/*F*_m_ values up to 88% of their initial values after 6 days following rewatering (Figure 2A). This was mainly achieved by shedding of almost all senescing leaves (Figure 2B). In contrast, mean *F*_v_/*F*_m_ of perishing plants continued to decrease to reach undetectable threshold of photosynthetic activity (*i.e.*, plants were completely senescing or decomposing; Figure 2B and Additional file 2: Figure S2). A clear separation of mean *F*_v_/*F*_m_ between surviving and perishing plants was visible four days after rewatering (Figure 2A).

### Computing and quantifying the heterogeneity of plant photosynthetic efficiency during growth and under severe water deficit

During SWD, *F*_v_/*F*_m_ values at the plant surface became heterogeneous, as illustrated by the changes in the mean and distribution of *F*_v_/*F*_m_ values (Figures 2A, B). We notably observed the establishment of multimodal distributions during SWD, reflecting the spatial variability of *F*_v_/*F*_m_ in the rosette (Figure 2B). To explore the heterogeneity of *F*_v_/*F*_m_ values during time course, we applied the Hartigan’s non-parametric significance test for unimodality [35-37]. As expected, the proportion of stressed plants showing multimodal distributions increased strongly after stress exposure (Figure 2C). Under WW conditions, the proportion of plants that displayed significant multimodal distributions also increased from < 10% to > 90% between 1 to 10 days after L4 stage. Stressed plants even displayed a slightly lower proportion of multimodal distributions compared to plants grown under WW conditions (Figure 2C).

**Figure 3:**
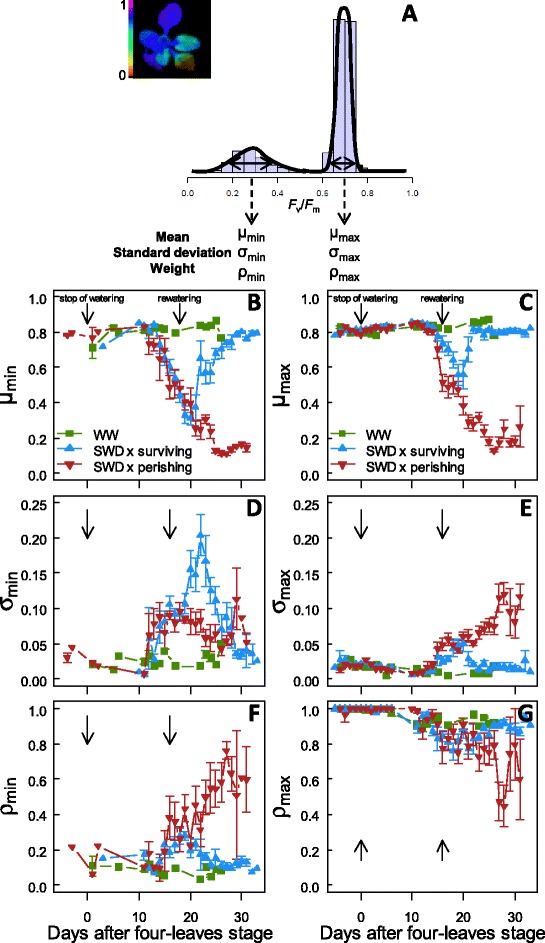
**Dynamics of the parameters describing the bimodal distributions. (A)** Example of a bimodal distribution composed of two clusters of pixels grouping in two modes. The higher mode (max; with the highest *F*_v_/*F*_m_ values) represents the healthiest parts of the rosette whereas the second mode (min; with the lowest *F*_v_/*F*_m_ values) represents damaged/senescing parts of the rosette. Each mode *i* of the mixture distribution of *F*_v_/*F*_m_ values is characterized by mean μ_i_, standard deviation σ_i_ and weight ρ_*i*_. **(B-G)** Dynamics of μ_max_ and μ_min_ of *F*_v_/*F*_m_ values, σ_max_ and σ_min,_ and, ρ_max_ and ρ_min_ in well-watered (WW) plants and under severe water stress (SWD; surviving and perishing plants) as a function of days after four-leaves stage (beginning of SWD) until bolting. Data are means (± SE) of 13–29 plants.

After distinguishing the plants that exhibited significant multimodal distributions, we used the REBMIX algorithm for finite mixture models [38] to characterize each mode *i* of the mixture of distributions of *F*_v_/*F*_m_ values (*i.e.*, mean μ_*i*_, standard deviation σ_*i*_ and weight ρ_*i*_) for each individual rosette. All distributions displaying multimodality were accurately represented by bimodal mixtures of normal distributions where the distributions are composed of two clusters of *F*_v_/*F*_m_ values grouping in two modes. The higher mode (maximum; μ_max_, σ_max_ and ρ_max_; with the highest *F*_v_/*F*_m_ values) represented the photosynthetically most efficient/healthiest parts of the rosette. The second mode (minimum μ_min_, σ_min_ and ρ_min_; with the lowest *F*_v_/*F*_m_ values) represented the least efficient or senescing parts of the rosette (Figure 3A). In case of unimodal distribution, the mode was considered as the single maximum mode.

For plants grown under WW conditions, each parameter was roughly constant during plant development (Figures 3B-G). In stressed plants, while μ_max_ essentially followed the same variation of whole-rosette mean *F*_v_/*F*_m_ (Figures 2A and 3C), μ_min_ decreased to reach very low values (μ_min_ = 0.24 ± 0.13 and 0.37 ± 0.17 for perishing and surviving plants, respectively; Figure 3B). Standard deviation σ_max_ progressively increased during SWD establishment. However, while σ_max_ of surviving plants recovered values similar to WW plants after rewatering, σ_max_ continued to increase in perishing plants (Figure 3E). By contrast, standard deviation σ_min_ increased more in surviving than in perishing plants, but recovered their initial value 13 days after rewatering (Figure 3D). In addition, the weight, *i.e.* the proportion, of the minimum mode ρ_min_ increased to a greater extent in perishing plants (and the weight of the maximum mode ρ_max_ decreased likewise) compared to surviving plants (Figures 3F, G).

**Figure 4:**
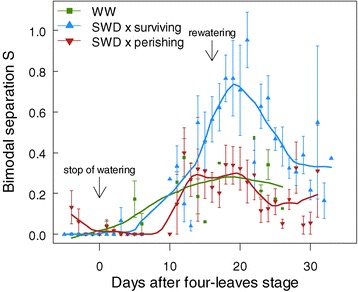
**Dynamics of spatial heterogeneity of whole-plant photosynthetic efficiency during development and severe water deficit.** Bimodal separation (S) of *F*_v_/*F*_m_ values of well-watered (WW) plants and stressed plants (SWD; surviving and perishing) as a function of days after four-leaves stage (beginning of SWD) until bolting. S = (μ_max_ - μ_min_) / 2 (σ_max_ + σ_min_) measures the distance between the modes and is superior to 1 essentially if the two modes do not overlap. Data are means (± SE) of 13–29 plants.

A quantification of the disparity between the two modes of a bimodal distribution, *i.e.* the heterogeneity of the values, is given by the ‘bimodal separation’ S = (μ_max_ - μ_min_) / 2(σ_max_ + σ_min_) [39]. S is roughly the distance between the two peaks, and S > 1 when the two modes do not overlap. Here, the *F*_v_/*F*_m_ heterogeneity across the plant increased regardless of soil water conditions during time course (Figure 4). However, S increased more in plants that survived the SWD than in others plants, whereas perishing plants had the same heterogeneity than those grown in WW conditions. A clear difference between S values of surviving and perishing plants was visible just before rewatering (Figure 4), *i.e.* four days earlier than mean *F*_v_/*F*_m_.

### Quantifying the effect of photosynthetic heterogeneity on whole-plant performance: description

Under SWD, S accurately represented the photosynthetic heterogeneity and allowed deciphering surviving and perishing plants. However, it failed to quantify the effect of photosynthetic heterogeneity on plant performance and stress tolerance, as shown by the overlap of S values between WW and perishing plants (Figure 4). This is because the deviation of both modes to the photosynthetic optimum is as important as the disparity between the two modes.

**Figure 5:**
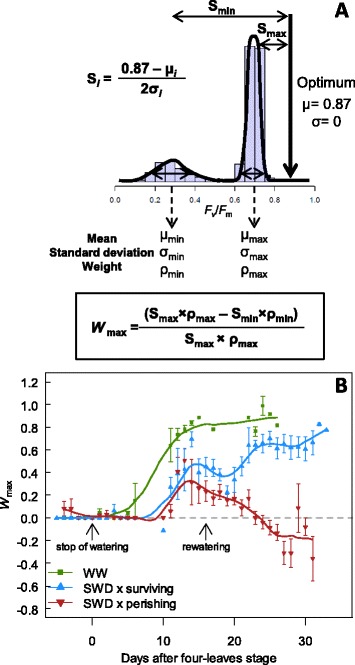
**Dynamics of the spatial efficiency of a photosynthetically heterogeneous plant (** ***W***_max_ **). (A)** Illustration of the mixture parameters in the case of a bimodal distribution. *W*_max_ is calculated as the proportional difference in the weighted bimodal separation of each mode (S_max_ and S_min_) to the theoretical optimum of photosynthetic performance (0.87, with standard deviation = 0), such as: *W*_max_ = (S_max_ × ρ_max_ - S_min_ × ρ_min_) / S_max_ × ρ_max_. *W*_max_ estimates the relative contribution of the most efficient/healthiest leaf regions to the whole-plant photosynthetic performance. **(B)** Dynamics of *W*_max_ of plants under well-watered (WW) and severe water deficit (SWD; surviving and perishing) conditions as a function of days after four-leaves stage (beginning of SWD) until bolting. Data are means (± SE) of 13–29 plants.

It was shown from energy conversion modelling of PSII that theoretical optimum of *F*_v_/*F*_m_ is about 0.87 in unstressed dark-adapted leaves [40,41]. However, a healthy plant displays a typical maximal mean *F*_v_/*F*_m_ = 0.83 [8,9] and shows considerable variation around the mean. The theoretical optimum would be reached if a plant exhibits a unimodal distribution of mean 0.87 and variance 0. Hence, the photosynthetic deviation of each mode *i* to the theoretical optimum can be estimated as the bimodal separation S_*i*_ such as S_*i*_ = (0.87 - μ_*i*_) / 2 σ_*i*_ (*i.e.*, S_max_ and S_min_; Figure 5A). High S_*i*_ represents low photosynthetic performance of the mode *i*. Then, the weighted deviation to the optimum, which measured the size-corrected performance of a given mode, was calculated as S_max_ **×** ρ_max_ and S_min_ **×** ρ_min_, for the maximum and the minimum modes, respectively (Figure 5A). To estimate the spatial efficiency of a photosynthetically heterogeneous plant to convert light energy into chemical energy (*W*_max_), we calculated the proportion of S_max_ **×** ρ_max_ (*i.e.*, the weighted deviation to the optimum of the most efficient leaf regions) in the distribution of *F*_v_/*F*_m_ values, as *W*_max_ = (S_max_ **×** ρ_max_ - S_min_ **×** ρ_min_) / S_max_ **×** ρ_max_ (Figure 5A). By definition, for a unimodal distribution *W*_max_ = 0 because there is no spatial heterogeneity (S_max_ **×** ρ_max_ = S_min_ **×** ρ_min_). Basically, increase or decrease in *W*_max_ indicates that the contribution of the most efficient/healthiest regions to the whole-plant photosynthetic performance is more or less important, respectively, than the contribution of the least efficient or senescing regions (note that *W*_max_ has a maximum value of 1). For a heterogeneous surface (*i.e.*, not in the first stages of plant development which display *W*_max_ = 0 because of unimodal distributions), *W*_max_ = 0 is assumed to be the compensation point, where the healthiest leaf regions compensate the negative effect of the less efficient leaf regions. Negative values of *W*_max_ appear when the contribution of senescing leaf regions is prevailing.

### Quantifying the effect of photosynthetic heterogeneity on whole-plant performance: applications

In plants grown in WW conditions, *W*_max_ increased progressively during development from 0 to *ca.* 0.85 (Figure 5B). This reflects the increase in the heterogeneity of whole-plant photosynthetic performance (*i.e.*, a switch from unimodality to bimodality) with a very low and negligible effect of the minimum mode compared to the maximum mode. In stressed plants, the increase of *W*_max_ was delayed and reduced (Figure 5B). In surviving plants, *W*_max_ started to decrease at 15 days after L4 stage, and recovered shortly (2 days) after rewatering. At bolting, surviving plants exhibited a *W*_max_ of *ca.* 0.65, *i.e.* 23% less than WW plants at the same developmental stage (Figure 5B). By contrast, in perishing plants, *W*_max_ started to decrease after 14 days following the L4 stage and became negative ten days later.

We used simple mathematical functions to model the dynamics of the parameters of the bimodal distributions in various stressing conditions, and simulate the associated variations of S and *W*_max_ (see Additional file 3). First, this simulation exercise was sufficient to reproduce what has been observed in this paper in plants grown under SWD that did not survive the stress. The parameters of these functions were then varied to simulate different scenarios of photosynthetic heterogeneity generated by different stress intensities. Our sensitivity analysis of *W*_max_ showed that it becomes as negative as (1) the rate of decrease in means and (2) the increase in proportion of damaged leaf regions, are high (*i.e.*, low stress tolerance, and/or diffuse stress effect, high stress intensity). Conversely, its decrease is delayed when the rates of decrease in means and rates of changes are low (*i.e.*, high stress tolerance, stress effects with high patchiness, and/or low stress intensity; see Additional file 3).

**Figure 6:**
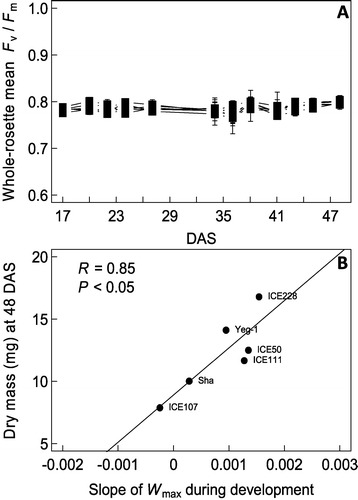
**Variation of** ***F***_v_ **/** ***F***_m_ **and relationship between** ***W***_max_ **and growth in six accessions of** ***A. thaliana*** **. (A)** Dynamics of whole-rosette mean *F*_v_/*F*_m_ as a function of days after stratification (DAS). Pots (n = 4) were manually watered three times per week to maintain good (non-stressing) soil moisture. **(B)** Relationship between the slope of *W*_max_ in the course of plant development and plant dry mass at 48 DAS. The accessions were collected from six different geographic origins (ICE107: South Italia; Sha: Kazakhstan; ICE111: South Italia; ICE50: Spain; Yeg-1: Caucasus; ICE228: South Tyrol). R: Pearson’s product–moment correlation coefficient.

To explore further the possible applications of *W*_max_, we performed the same analysis on two other datasets. First, we used an independent dataset (not generated with the PHENOPSIS platform) to explore the genetic variability in photosynthetic performance in six accessions of Arabidopsis from contrasted geographic locations. The plants displayed little variation during plant development in mean *F*_v_/*F*_m_ values (Figure 6A). However, we observed an increase in photosynthetic heterogeneity S and *W*_max_ during plant development (see Additional file 4: Figure S3). We calculated the increase in *W*_max_ during development as the slope of the relationship between *W*_max_ and plant age. Interestingly, we found that 72% of the variability in plant dry mass at 48 days after stratification (DAS) was explained by the variation of *W*_max_ between 17 and 48 DAS (*P* < 0.05; R = 0.85; Figure 6B).

Second, we investigated the effect of soil inoculation with *Phyllobacterium brassicacearum* STM196 strain, a plant growth-promoting rhizobacteria (PGPR) that improves plant tolerance to moderate water deficit [42] and also increases plant survival under SWD [19]. Bresson *et al*., 2014 [19] showed that STM196-inoculated surviving plants also exhibit a higher growth recovery after rewatering, leading to a higher plant biomass than non-inoculated plants [19]. Here, we showed that STM196-inoculation induced a faster and higher increase in *W*_max_ than non-inoculated plants from 2 days after rewatering (Additional file 5: Figure S4). This suggests that the positive effects of STM196 on growth recovery, biomass production and plant survival may be related to its effects on whole-plant photosynthetic heterogeneity.

## Discussion

Analysing the effects of environmental conditions on plant growth, survival and yield requires massive, rapid and non-invasive tools to track changes in plant performance. Non-invasive ChlF imaging has been developed to give insights into plant photosynthetic capacities and explore the ability of plants to tolerate various environmental stresses (*e.g.*, [8,16,43]). Most often the mean values of various indices of ChlF, including the widely used *F*_v_/*F*_m_, of an organ or a plant is used to characterize the response to a stressor (*e.g.*, [8,11]). However, a ChlF image is composed of a panel of pixels in a given range (*F*_v_/*F*_m_ = [0; 1]). Hence, using mean values does not give a clear clue of the disparity of values that corresponds to contrasted physiology. Heterogeneity in the photosynthetic capacities of plants has been observed but rarely quantified in responses to a wide variety of external stimuli (*e.g.*, [10,16,25-32]). For instance, the establishment of *F*_v_/*F*_m_ heterogeneity in response to stress has been described by sampling small areas on the leaf surface [10,29,30], visual inspection of the shape of distributions [25-29,32], or by *F*_v_/*F*_m_ clustering [16]. However, this is prone to large variations depending on the species, experimenter and stress. ChlF heterogeneity is often admitted but its standardized, objective and reproducible quantification is still lacking. For instance, previous methods used threshold-based analysis to quantify the area and progression of senescence or damages [8,16]. Here, we proposed a method to quantify (1) the heterogeneity of *F*_v_/*F*_m_ values at whole-plant level (S) and (2) the spatial efficiency of a photosynthetically heterogeneous plant (*W*_max_). Although we applied our method to measurements of *F*_v_/*F*_m_ in the Arabidopsis rosette under a severe water deficit scenario, we argue that the approach can be used with other ChlF parameters (*e.g.*, ΦII, NPQ) as well as in response to other stressing conditions that induce variations of the physiological status.

### Photosynthetic heterogeneity is intrinsic to the development of plants

Our results showed that the distribution of *F*_v_/*F*_m_ values shifted from unimodal to bimodal distributions both under WW and SWD conditions, and this despite a constant mean *F*_v_/*F*_m_ in WW plants. This result indicates that heterogeneity in photosynthetic efficiency (*i.e.*, the increase in the proportion of bimodal *F*_v_/*F*_m_ distributions) does not appear only under stress but is intrinsic to the development of plants. Importantly, S and *W*_max_ in WW plants also significantly increased during development. It therefore indicates that, even in the absence of visible senescence, (1) there were leaf regions exhibiting lower *F*_v_/*F*_m_, (2) low-efficiency leaf regions increased during development and, (3) the contribution of these latter was minor on whole-plant photosynthetic performance under WW conditions. There might be different sources of photosynthetic heterogeneity. First, at the whole-plant level, photosynthetic heterogeneity in plants might be caused by age-induced leaf senescence, *i.e.* by visible and non-visible cell death and nutrient remobilization, notably on the edges of the oldest leaves. In addition, the increase in the size of leaf veins with increasing leaf size can also induce a decrease in the mean *F*_v_/*F*_m_, as well as an increase in *F*_v_/*F*_m_ heterogeneity. Second, at the sub-cellular level, some of PSII centres are inactive to linear electron transport. Functional PSII heterogeneity is for instance expected since 70-80% of PSII are located in the stacked grana region and the remaining PSII are located in the stroma-exposed region of the thylakoid membrane [44-47].

We also showed that the variation in photosynthetic heterogeneity might be a key trait related to plant growth, as suggested by the significant correlation between the increase in *W*_max_ during development and biomass in six contrasted Arabidopsis accessions and despite no distinct differences in the mean *F*_v_/*F*_m_ between genotypes. The analysis of the distributions of *F*_v_/*F*_m_ values, as proposed with S and *W*_max_, allows the quantification of the whole-plant heterogeneity and may be more informative than the whole-plant mean value to investigate changes during plant development and genetic variation in plant performance.

### The indicators of photosynthetic heterogeneity (S and *W*_max_) are linked to plant tolerance to severe water deficit

Our analysis revealed that SWD affected the establishment of the intrinsic heterogeneity in plants during development. The heterogeneity of *F*_v_/*F*_m_ values (quantified by S) across the rosette increased differently depending on the state of the plants. Importantly, S was a more sensitive indicator of the plant physiological status than the mean *F*_v_/*F*_m_. Indeed the mean *F*_v_/*F*_m_ was stable during the first 14 days in stressed plants, while a strong photosynthetic heterogeneity was already present (Figures 2A and 4). S allows the discrimination between surviving and perishing plants earlier, *ca*. four days, than the whole-rosette mean *F*_v_/*F*_m_. The lag time before recovery was also shorter in S values than the mean *F*_v_/*F*_m_.

Surprisingly, surviving plants displayed a higher increase of S than the others plants during stress establishment, and perishing plants exhibited S dynamics similar to plants grown under WW conditions. This did not reflect the lower absolute values of *F*_v_/*F*_m_ in perishing plants. The higher photosynthetic heterogeneity in surviving plants can be explained by the establishment of a gradient of *F*_v_/*F*_m_ values from the tip to the base in the oldest leaves, often observed under water stress [10] and with high *F*_v_/*F*_m_ values in the youngest leaves (as suggested in this study, see Figure 2). The analysis of the different parameters of bimodal distributions shows that SWD did not induce a global decrease of *F*_v_/*F*_m_, but plants rather maintained leaf regions with near-optimum *F*_v_/*F*_m_ and sacrificed other leaf regions. Moreover, plant survival to SWD was associated to a large variability in *F*_v_/*F*_m_ of the most damaged/senescing leaves; but to a low variability in the healthiest leaves or leaf regions (Figure 3). After rewatering, we showed that surviving plants recovered optimal mean *F*_v_/*F*_m_ values with decreasing S, by loss of senescing leaves and/or by development of new leaves with optimal *F*_v_/*F*_m_. This is in accordance with the survival strategy of plants aiming at recycling and reallocating resources from the oldest or senescing leaves to active growing organs [23]. On the contrary, perishing plants displayed a decrease of *F*_v_/*F*_m_ values in their oldest but also youngest leaves, resulting in a lower and constant value of bimodal separation S across the rosette. Perishing plants thus presented the same heterogeneity than well-watered plants.

However, the contribution of the most efficient leaf regions was more negatively impacted in the perishing plants. In surviving plants, *W*_max_ decreased during stress establishment and recovered rapidly after rewatering. This indicated that the contribution of the healthiest leaf regions was sufficient to compensate the negative effects of senescence and to support plant survival. By contrast, in perishing plants, *W*_max_ constantly decreased and reached negative values, reflecting that the contribution of low-efficiency leaf regions on whole-plant photosynthetic performance was too important and led to plant death. We also showed that increase in *W*_max_ is related to the improvement of plant survival and biomass production upon inoculation with a PGPR that affects photosynthesis in plants [42]. We argue that *W*_max_ could be a good indicator to predict plant survival under water stress, and presumably in response to other stresses.

The results of the sensitivity analysis (Additional file 3) showed that *W*_max_ is specifically sensitive to changes in the proportion of damaged leaf regions and in the lag time, *i.e.* the time before the appearance of the first symptoms. By contrast, S is independent of the proportion of leaf damaged but strongly impacted by the dynamics of the means. This simulation analysis suggests that the variation of *W*_max_ during stress (specifically, the rate of decrease and the time to reach negative values) can be a good indicator of stress tolerance in response to many environmental stresses. We argue that the parameters of the mathematical functions used to model the dynamics of mixture distributions could be used as useful tolerance/sensitivity indices. Additional work is however needed to implement physiological hypotheses under the spatio-temporal dynamics of the mixture parameters.

Together, our results showed that the dynamics of S and *W*_max_ allow quantifying photosynthetic heterogeneity and its relationship with plant performance, during plant development and under stress. Although the variation in mean *F*_v_/*F*_m_ is informative about overall changes in plant performance, we showed that the modifications of *F*_v_/*F*_m_ distributions are not accurately tractable by the modifications of mean *F*_v_/*F*_m_, or other threshold-based methods. For example, in comparison to Woo *et al.* [8] who used a 33% threshold for a mean *F*_v_/*F*_m_ = 0.80 to discriminate surviving to perishing plants, here we showed that the bimodal separation S allowed discriminating plants four days earlier than mean *F*_v_/*F*_m_. Moreover, the quantitative measurement of photosynthetic heterogeneity can be followed, repeated and modelled in the course of plant development. For instance, negative values of *W*_max_ indicated death for individual plants; this may represent a powerful tool to screening plants to water stress. Our study highlights that the management of the spatial photosynthetic heterogeneity may be key to plant survival, and that the *F*_v_/*F*_m_ heterogeneity is a sensitive measure of plant responses to the environment. Further studies will undoubtedly improve our capacity to predict plant tolerance, including survival, to different stressing environmental conditions using the indices of photosynthetic heterogeneity.

## Conclusion

Heterogeneity in photosynthetic performance has implications for overall plant performance. In this study, we characterized the heterogeneity of chlorophyll fluorescence in leaves impacted by severe water deficit. We used a quantitative measure of (1) the heterogeneity of photosynthetic efficiency S, and (2) the spatial efficiency of a photosynthetically heterogeneous plant, *W*_max_. S and *W*_max_ gave a more accurate indication of the dynamics of leaf senescence or damages induced by water deficit than the whole-plant mean *F*_v_/*F*_m_. For instance, they allowed an earlier discrimination between surviving and perishing plants. S and *W*_max_ were also strongly related to the genetic variability of growth between six contrasted accessions of Arabidopsis. Together our analyses suggest that S and *W*_max_ could be useful indicators of plant responses to their abiotic and biotic environments. Other studies are now needed to explore further the physiological causes and implications of the quantitative variations of photosynthetic heterogeneity and then improve our ability to predict plant response to the environment.

## Methods

### Phenotyping platform PHENOPSIS

The PHENOPSIS platform [33] includes three automatons set up in growth chambers strictly controlled for environmental conditions. In each chamber, an automated arm is equipped with a balance (XB620C; Precisa, Dietikon, Zurich, Swiss) and a complete system of irrigation (A1645-6; Electro finish, Saint-Egrève, France) to accurately weigh and irrigate up to 504 *Arabidopsis thaliana* plants in individual pots. The arm is also equipped with multiple devices for non-destructive acquisition of plant phenotypic data such as Charge Coupled Device (CCD) cameras or fluorescence sensors [1]. Light is provided by a bank of cool-white fluorescent tubes (36 W T8 Grolux, 120 cm; Sylvania) and quartz metal halide lamps (HPI-T Plus 400 W 645 E40; Philips). Light intensity is measured continuously at plant height, using a light sensor over the waveband of 400–700 nm (SKP215; Campbell Scientific, Logan, USA). Air temperature and relative humidity are measured every 20 s (HMP45C-L; Campbell Scientific). All measurements of temperature, light intensity and relative humidity are averaged and stored every 600 s in a data-logger (CR10X; Campbell Scientific) with data-logger support software (Loggernet V4; Campbell Scientific). The climatic regulation of the growth-chambers is controlled by Loggernet software allowing the control of the desired environment by employing an air drier or a water sprayer to modify air humidity, an air-cooler or a heater to modify air temperature.

### Plant material, growth conditions and irrigation treatments

The experimentations performed in the PHENOPSIS automaton used *A. thaliana* (L.) Heynh, accession Columbia-0. Five seeds were sown at the soil surface in 250 cm^3^ cylindrical pots (10 cm high, 6 cm diameter) filled with a damped mixture (1:1, v:v) of loamy soil and organic compost Neuhaus N2 (see Additional file 6: Table S1 for soil chemical properties). Initial soil water content was controlled during pot filling by determining soil fresh weight (FW_soil_) and soil dry weight (DW_soil_, after 5 days at 80°C) every ten pots. Soil relative water content was calculated as RWC_soil_ = (FW_soil_ – DW_soil_) × 100 × DW_soil_^−1^. Subsequent changes in pot weight were attributed to a change in soil water status. The pots were kept in the dark for 2 days and were damped with sprayed deionised water three times a day until germination. Then, plants were cultivated under 12 h day length (180 μmol m^−2^ s^−1^ photosynthetic photon flux density, at plant height). During germination phase (7 days), air temperature was set to 20°C day and night, and air relative humidity was adjusted in order to maintain constant water vapour pressure deficit (VPD) at 0.6 kPa. Then, plants were grown at 20/17°C day/night and 0.8 kPa of VPD. Just before the beginning of water stress, seedlings of similar sizes and developmental stages were selected and were thinned to one to four plants per pot. Each pot was daily weighed and watered with a modified one-tenth-strength Hoagland solution [48] to reach the target RWC_soil_. RWC_soil_ was maintained at 0.35 g H_2_O g^−1^ dry soil in the WW treatment (35%). SWD was started at L4 stage by stopping irrigation to decrease progressively RWC_soil_ to reach 0.06 g H_2_O g^−1^ dry soil (6%). After RWC_soil_ = 6% g H_2_O g^−1^ dry soil, irrigation was resumed by adding a daily constant volume of nutritive solution to reach the WW soil condition level, and was then maintained until final harvests at first flower open (stage 6.00; [34]). Soil water potential was determined by using a potentiometer (WP4-T dewpoint meter, Decagon Devices, Pullman, WA 99163, USA) during the soil drying.

An independent experiment was performed in the Max Planck Institute for Developmental Biology (Weigel lab, Tübingen, Germany) on six natural accessions from contrasted geographic origins: ICE107 (South Italia), ICE111 (South Italia), ICE228 (South Tyrol), ICE50 (Spain), Sha (Kazakhstan), Yeg-1 (Caucasus). Each accession was grown in four replicates. Five to ten seeds were sown at the soil surface of each pot and stratified during 2 days in the dark at 4°C. Plants were then grown at 16°C and under 8 h day length. At L4 stage, only one plant per pot was kept and grown until 48 DAS. Pots were manually watered 3 times a week to maintain good soil moisture. *F*_v_/*F*_m_ was measured every 2–3 days from 17 to 48 DAS (using the same ChlF imaging system as in the PHENOPSIS automaton described below). At 48 DAS, rosettes were harvested, dried at 65°C for 4 days and weighed.

### High resolution of chlorophyll fluorescence imaging

#### Acquisition of chlorophyll fluorescence images

ChlF measurements were performed using Imaging-PAM chlorophyll fluorometer and ImagingWin software application (ver. 2-45d, Heinz Walz GmbH) connected with PHENOPSIS automaton. ImagingWin software is driven by Optima PLC (ver. 2–14, build v323, by Optimalog SARL; Saint-Cyr-sur-Loire, France) that allows the automatic movement of Imaging-PAM implemented on the arm of robot (Figure 1A) and the ChlF measurement of each pot with identical settings. The measuring system consists of a 2/3″ Gigabit Ethernet IMAG K6-CCD camera (Manta, G-145B, ASG Allied Vision Technologies GmbH, Stadtroda, Germany), 1392 × 1040 pixel primary resolution with enhanced sensitivity by 4-pixel-binning resulting in 640 × 480 pixel images, coupled to an objective lens (F1.4/f = 12.5 mm; Cosmicar-Pentax, Hamburg, Germany) with a detector filter (RG665, 3 mm) and a short-pass interference filter (λ < 770 nm). Imaging-PAM is equipped with a powerful array of 44 high-power Luxeon LEDs for fluorescence excitation and actinic illumination with blue light (450 nm) as well as assessment of absorbed photosynthetically active radiation with the help of red light (650 nm) and near-infrared (NIR)-light (780 nm). The ChlF imaging system was equipped by a conic, black, metal shading hood of 21.5 cm height wherein the pot was lift up by the balance controlled by a hydraulic cylinder (Figure 1A). This system allows avoiding the illumination of neighbouring plants and achieving the optimal working of 18.5 cm distance from the LEDs resulting in an imaged area of approximately 9 x 12 cm with +/− 7% maximal deviation of intensity from the mean value. The image acquisition takes 30 s per plant, and different files are generated such as .PIM files (which are only usable by ImagingWin software) and .CSV files, which contain requested parameters such as *F*_0_, *F*_m_ and *F*_v_/*F*_m_ averaged on a region of interest. Two images of ChlF acquisition are also generated: *.JPEG files which give a representation of the image acquired in false colour (from black pixel values (0) through red, yellow, green, blue to purple (ending at 1)) and *.TIFF files with contain different stacks of fluorescence parameters (see below).

#### Measurement of maximum efficiency of PSII (*F*_v_/*F*_m_)

The ChlF measurement was initiated by exposing dark-adapted leaf to measuring light pulses (1 Hz frequency, Intensity 2) for determination of *F*_0_. The *F*_m_ level of fluorescence is recorded during a saturating pulse (Si 9, width 800 ms). *F*_v_/*F*_m_, calculated as (*F*_m_-*F*_0_)/*F*_m_, provides the maximum quantum yield of PSII (*i.e.*, photosynthetic efficiency). In the study, *F*_v_/*F*_m_ was daily performed, from the two first leaves to bolting, on dark-adapted plants (8–12 h of dark), under WW and SWD conditions.

### Data extraction of photosynthetic efficiency and rosette expansion during time course

We developed an ImageJ macro “PHENOPSIS-Fluo” to semi-automatically extract whole-rosette *F*_v_/*F*_m_ pixel values and total leaf area from the generated TIFF files (containing *F*_0_ and *F*_m_ pixel values, and NIR images). The analysis of ChlF images starts with image segmentation from NIR pictures, *i.e.* the automatic separation of the region of interest (here the rosette) from the background. Then, by subtracting and dividing *F*_0_ and *F*_m_ stacks, [(*F*_m_ – *F*_0_)/*F*_m_], the macro generates an image of *F*_v_/*F*_m_ pixel values, which are represented in the 255 greyscale (0 and 255 corresponding to the minimum and maximum pixel values of the selection, respectively). The macro gives the whole-rosette *F*_v_/*F*_m_ mean and the list of pixel values across the plant. Projected area of the rosette (RA_*proj*_) was also determined from plant selection. Different plants in the same pot can be independently measured. Whole-rosette mean *F*_v_/*F*_m_ extracted with the macro “PHENOPSIS-Fluo” was highly correlated with the mean of the rosette extracted from the commercial software ImagingWin (*R*^2^ = 0.98; Additional file 7: Figure S5). The “PHENOPSIS-Fluo” macro is available on the PHENOPSIS website (http://bioweb.supagro.inra.fr/phenopsis/MacroImageJ.php).

Under SWD conditions, growth dynamics of surviving plants were modelled as a sigmoid curve fitted following RA_*proj*_ = *a* / [1 + exp-[(*d*-*a*/2)/*b*]] where *a* is the maximum area, and *d* is the number of days after L4 stage. Under WW conditions, an exponential curve was fitted such as RA_*proj*_ = exp (*a* – *b* × *d*).

### Statistical analyses and modelling procedures

All analyses were performed using R 3.1[49]. Comparisons of mean trait values between treatments were performed with Kruskal-Wallis non-parametric tests. Survival percentage was calculated as the proportion of surviving plants at the end of experiment compared to initial number of plants. Plant survival was analysed by Chi^2^ tests. Non-parametric significance test for unimodality, Hartigan’s dip test (R package ‘dip test’ [35-37]) was used to identity multimodal distribution, with *p*-value < 0.01 as the significance threshold for departure from unimodality. Then, the REBMIX algorithm for finite mixture models ([38]; R package ‘rebmix’) as used to characterize each mode *i* of the bimodal distributions of *F*_v_/*F*_m_ values (*i.e.*, mean μ_*i*_, standard deviation σ_*i*_ and weight ρ_*i*_), using the Bayesian Information Criteria (BIC).

We developed a modelling procedure of the temporal dynamics of the means, standard deviations and proportions (weights) of the mixture distributions. We then simulated the variation of these parameters and, subsequently, the variation of S and *W*_max_ (Additional file 3).

All meteorological and phenotypic data, ChlF files and images, R scripts are available in the PHENOPSIS web site (http://bioweb.supagro.inra.fr/phenopsis/) and database [50].

## Additional files

Additional file 1: Figure S1. Soil water potential during soil drying. Soil water potential was determined using a potentiometer (WP4-T dewpoint meter, Decagon Devices, Pullman, WA 99163, USA) during soil drying (from 0.35 to 0.06 g H_2_O g^−1^ dry soil). Additional file 2: Figure S2. Representations of vegetative rosettes in *F*_v_/*F*_m_ false colour (from black pixel values (0) through red, yellow, green, blue to purple (ending at 1)) under well-watered (WW) conditions and under severe water stress (SWD; surviving and perishing plants) during time courses. Additional file 3:
**Modelling the spatial heterogeneity of photosynthetic efficiency under stress.**
Additional file 4: Figure S3. Spatial heterogeneity S of whole-plant *F*_v_/*F*_m_ and *W*_max_ in six accessions of *A. thaliana*. (A) Bimodal separation S and (B) the spatial efficiency of a photosynthetically heterogeneous plant *W*_max_ as a function of days after stratification (DAS). The six accessions were collected from different geographic origins (ICE107: South Italia; Sha: Kazakhstan; ICE111: South Italia; ICE50: Spain; Yeg-1: Caucasus; ICE228: South Tyrol). Additional file 5: Figure S4.
*W*_max_ under biotic and abiotic interaction: effect of inoculation by a plant growth promoting rhizobacteria (PGPR) under severe water stress. *W*_max_ in non-inoculated plants (NI; solid lines) and inoculated plants with the PGPR *Phyllobacterium brassicacearum* (STM196; dashed lines) under well-watered (WW) conditions and severe water stress (SWD; surviving and perishing) as a function of days after four-leaves stage (beginning of SWD) until bolting. Additional file 6: Table S1. Soil chemical properties of the compost (Neuhaus N2), soil and two mixtures of both. Mixture 1 was sampled before experimentation and mixture 2 was sampled after experimentations. nd: not determined. Soil analysis was performed by ALFA Agricultural Service and Research Building, Soil Testing Laboratory of Auburn University. Additional file 7: Figure S5. Correlation between extracted whole-rosette mean *F*_v_/*F*_m_ with the macro “PHENOPSIS-Fluo” and extracted from CSV files from ImagingWin software. *R* ^2^ is the Pearson’s coefficient of correlation between 164 rosettes analysed with both the PHENOPSIS-Fluo macro and the ImagingWin software.

## Abbreviations

ChlF: Chlorophyll fluorescence
PSII: Photosystem II
Q_A_: Quinone A, the primary stable electron acceptor of PSII centres
*F*_v_/*F*_m_: Maximum quantum efficiency of PSII photochemistry (photosynthetic efficiency)
*F*_0_: Minimal fluorescence emission of a dark-adapted plant
*F*_m_: Maximum fluorescence emission after a short pulse of a saturating light
*F*_v_: Variable fluorescence from dark-adapted plant
S: Spatial heterogeneity of *F*_v_/*F*_m_
*W*_max_: Spatial efficiency of a photosynthetically heterogeneous plant
SWD: Severe water deficit
L4: Four-leaves stage
RWC_soil_: Soil relative water content
WW: Well watered condition
DAS: Days after stratification
PGPR: Plant growth-promoting rhizobacteria
CCD: Charge Coupled Device
FW_soil_: Soil fresh weight
DW_soil_: Soil dry weight
VPD: Water vapour pressure deficit
NIR: Near-infrared
RA_*proj*_: Projected area of the rosette
