# Supplementary figures and images for: Quantifying spatial heterogeneity of chlorophyll fluorescence during plant growth and in response to water stress
Source: Plant Methods. 2015 Mar 26;11:23. doi: 10.1186/s13007-015-0067-5 (PMC4394423; doi:10.1186/s13007-015-0067-5)

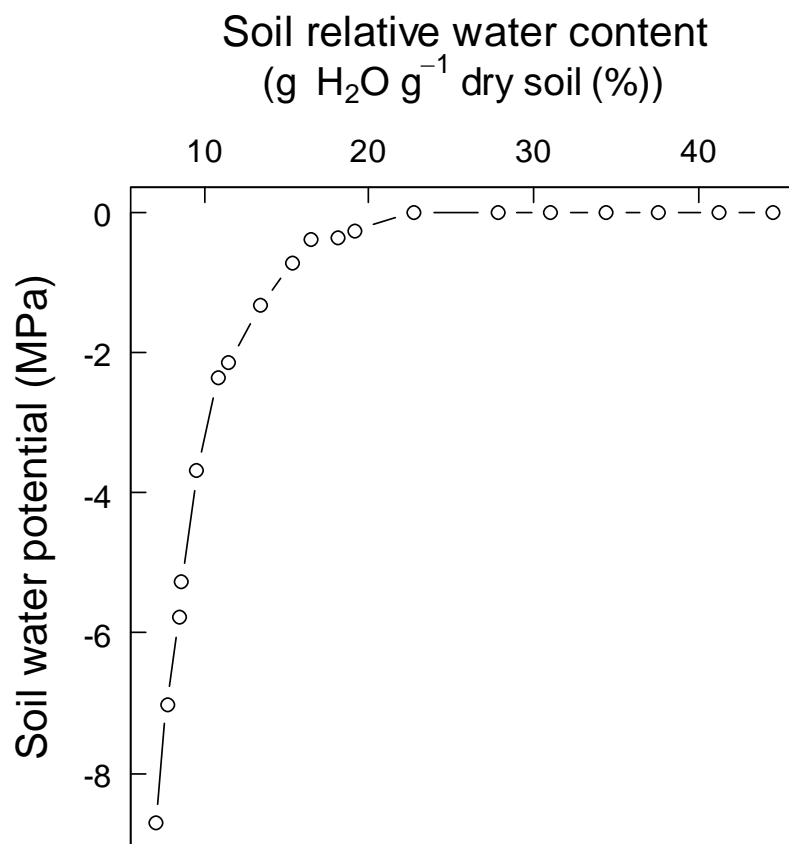

Supplement: Additional file 1: Figure S1. — Soil water potential during soil drying. Soil water potential was determined using a potentiometer (WP4-T dewpoint meter, Decagon Devices, Pullman, WA 99163, USA) during soil drying (from 0.35 to 0.06 g H2O g−1 dry soil). [file 13007_2015_67_MOESM1_ESM.pdf]

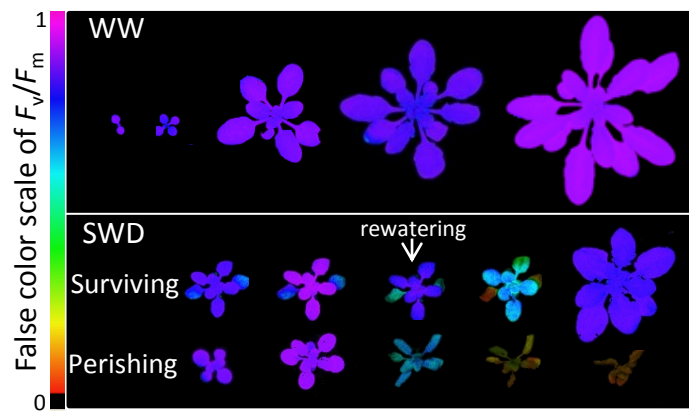

Supplement: Additional file 2: Figure S2. — Representations of vegetative rosettes in F v/F m false colour (from black pixel values (0) through red, yellow, green, blue to purple (ending at 1)) under well-watered (WW) conditions and under severe water stress (SWD; surviving and perishing plants) during time courses. [file 13007_2015_67_MOESM2_ESM.pdf]

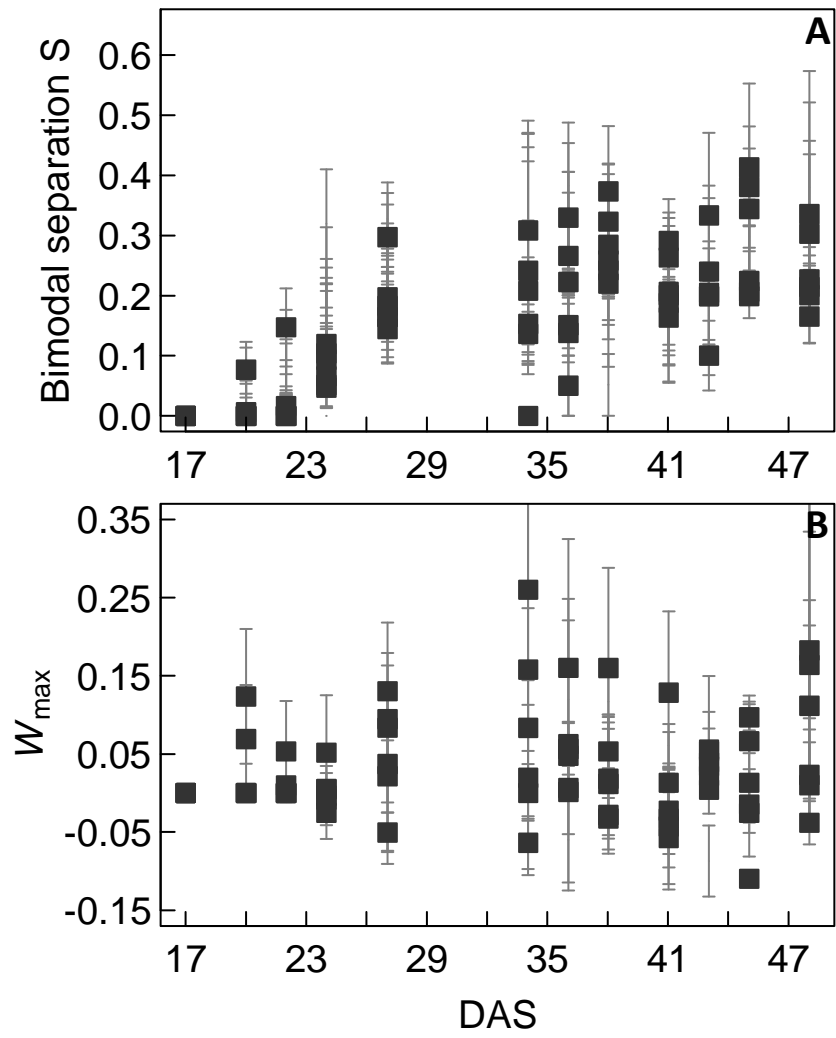

Supplement: Additional file 4: Figure S3. — Spatial heterogeneity S of whole-plant F v/F m and W max in six accessions of A. thaliana. (A) Bimodal separation S and (B) the spatial efficiency of a photosynthetically heterogeneous plant W max as a function of days after stratification (DAS). The six accessions were collected from different geographic origins (ICE107: South Italia; Sha: Kazakhstan; ICE111: South Italia; ICE50: Spain; Yeg-1: Caucasus; ICE228: South Tyrol). [file 13007_2015_67_MOESM4_ESM.pdf]

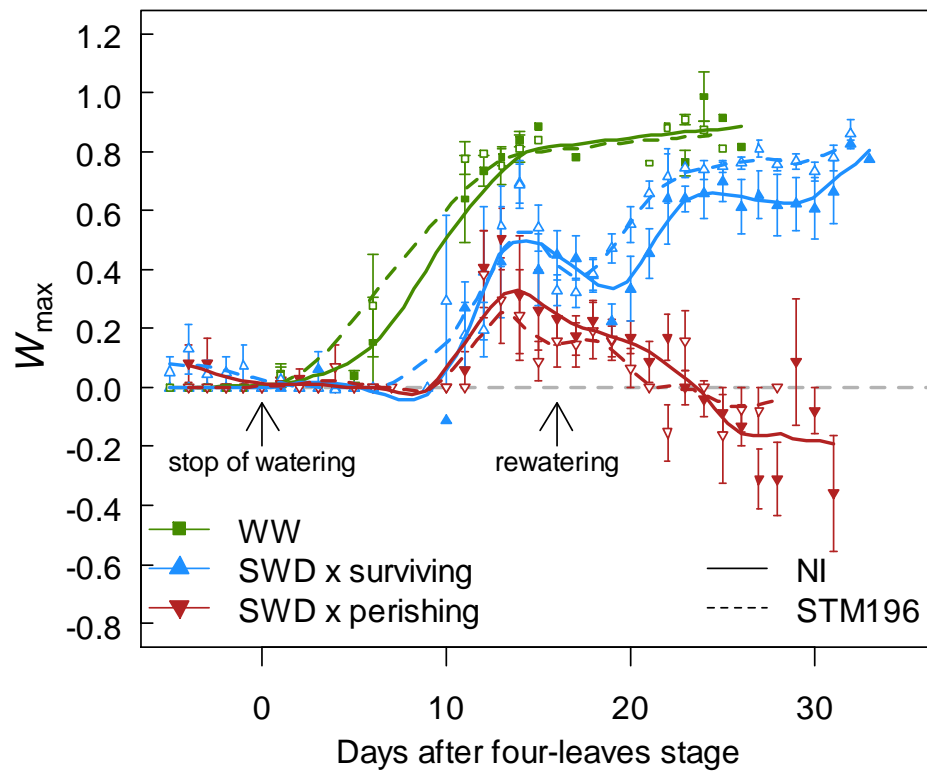

Supplement: Additional file 5: Figure S4. — W max under biotic and abiotic interaction: effect of inoculation by a plant growth promoting rhizobacteria (PGPR) under severe water stress. W max in non-inoculated plants (NI; solid lines) and inoculated plants with the PGPR Phyllobacterium brassicacearum (STM196; dashed lines) under well-watered (WW) conditions and severe water stress (SWD; surviving and perishing) as a function of days after four-leaves stage (beginning of SWD) until bolting. [file 13007_2015_67_MOESM5_ESM.pdf]

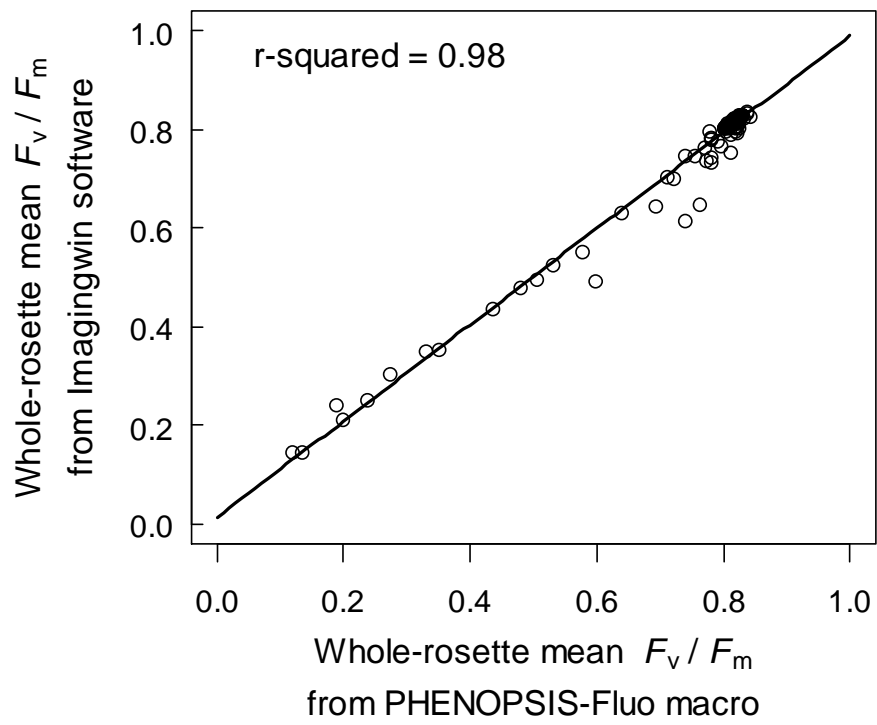

Supplement: Additional file 7: Figure S5. — Correlation between extracted whole-rosette mean F v/F m with the macro “PHENOPSIS-Fluo” and extracted from CSV files from ImagingWin software. R 2 is the Pearson’s coefficient of correlation between 164 rosettes analysed with both the PHENOPSIS-Fluo macro and the ImagingWin software. [file 13007_2015_67_MOESM7_ESM.pdf]
