# Supplementary material for: Quantifying spatial heterogeneity of chlorophyll fluorescence during plant growth and in response to water stress
Source: Plant Methods. 2015 Mar 26;11:23. doi: 10.1186/s13007-015-0067-5 (PMC4394423; doi:10.1186/s13007-015-0067-5)
Supplement: Additional file 3: — Modelling the spatial heterogeneity of photosynthetic efficiency under stress. [file 13007_2015_67_MOESM3_ESM.pdf]

Modelling the spatial heterogeneity of photosynthetic efficiency under stress

This is the Additional file 7 of the paper by Bresson *et al.* (*Plant Methods*).

Several abiotic or biotic factors, including abiotic stresses (water deficit, high temperature, freezing, salinity) and pathogens, can contribute to heterogeneous photosynthetic performance at a leaf or whole-plant levels due to their effects on plant physiology. Chlorophyll fluorescence (ChlF) imaging is among the existing imaging techniques (*e.g.* thermo-imaging, hyperspectral imaging) that provide indicators of a leaf or a plant photosynthetic performance.

In this paper by Bresson *et al.*, we observed that a severe water deficit caused drastic changes in the heterogeneity of photosynthetic efficiency, as measured by ChlF imaging on plants of *Arabidopsis thaliana* ecotype Col-0. Precisely, the effects of water deficit rapidly translated into bimodal Gaussian distributions of the maximum quantum efficiency of PSII photochemistry (F_v/F_m). This was due to an increase of leaf areas (beginning at leaf tips) with reduced photosynthetic efficiency as stress progressed whereas other parts remained healthy for a longer time. In perishing plants (60% of the plants) the whole leaf area of the rosette reached null values of F_v/F_m and plants failed to recover after rewatering.

In order to quantify and analyse the heterogeneity of photosynthetic performance at the leaf and whole-plant levels, we developed a statistical procedure that first identify mixture distributions of pixel values (here given by F_v/F_m) and extract the parameters of multi-modality, limited here to two modes (*i.e.* bimodality). Then, the temporal dynamics of the distribution is a mixture of two Gaussian distributions with means $\mu(t)_1$ and $\mu(t)_2$, standard deviations $\sigma(t)_1$ and $\sigma(t)_2$ and weights $\rho(t)_1$ and $\rho(t)_2 = 1 - \rho(t)_1$ that change as a function of time t.

From the parameters of bimodal mixture distributions we calculated two indices of heterogeneity: S, the bimodal separation, and W_{max}, the spatial efficiency of a photosynthetically heterogeneous plant. In the paper we showed that these indices allowed the comparison of the dynamics of photosynthetic heterogeneity among genotypes and in response to stress.

Here, in order to improve the presentation of the method and of its potentialities, we performed a simulation exercise to analyse the sensitivity of both S and W_{max} to changes in the dynamics of the bimodal parameters. Although further work is needed to improve the simulation process that could explicitly include spatial dynamics, functional hypotheses of variation of the parameters, or stress recovering, we argue that the parameters used to simulate the dynamics of the bimodal distributions could provide useful tolerance/sensitivity indices.

Modelling the temporal dynamics of mixture distributions

Temporal dynamics of means

The temporal dynamics of the means of the two distributions $\mu(t)_i$ were modelled as:

$$\mu(t)_i = \begin{cases} \mu_{max}, & if\ t < \delta_{\mu i}, \\ (\mu_{max} - \mu_{min}) \times e^{-\frac{(t-\delta_{\mu i})^2}{2(\theta_{\mu i}/8)^2}} + \mu_{min}, & otherwise, \end{cases} \quad \theta_{\mu i} \geq 0.$$

$\mu(t)_i$ is monotone, decreasing, and tends to μ_{min} when time tends to infinity and satisfies $0 \leq \mu(t)_i \leq \mu_{max}$ (see Figure 1a). Here, $\mu_{max} = 0.83$, the observed maximum F_v/F_m value. $\delta_{\mu i}$ is the lag time before $\mu(t)_i$ begins to decrease and $\theta_{\mu i}$ is the parameter that inversely affects the decreasing rate.

Temporal dynamics of standard deviations

The temporal dynamics of the standard deviations of the two distributions $\sigma(t)_i$ were modelled as an exponential function approaching the Normal distribution in the form:

$$\sigma(t)_i = a_i b_i^{(t-(c_i+\delta_{\sigma i}))^2} + \sigma_{min\ i}$$

Parameter a controls the height (y-value) of the maximum σ value; parameters c and δ_σ control the location (x-value) of the maximum (or minimum). $\sigma_{min\ i}$ controls the minimum standard deviation (see Figure 1b). $\sigma(t)$ is a straight line for $b = 1$, as used in the simulations presented in this document.

Temporal dynamics of weights of the distributions

The temporal dynamics of the weights of the distributions $\rho(t)_i$, were modelled as:

$$\rho(t)_1 = \begin{cases} \rho_{max}, & if\ t < \delta_\rho, \\ (\rho_{max} - \rho_{min}) \times e^{-\frac{(t-\delta_\rho)^2}{2(\theta_\rho/8)^2}} + \rho_{min}, & otherwise, \end{cases} \quad \theta_\rho \geq 0, 0 \geq \rho_{max} \leq 1, 0 \geq \rho_{min} \leq 1.$$

$$\rho(t)_2 = 1 - \rho(t)_1.$$

$\rho(t)_i$ is monotone, decreasing, and tends to ρ_{min} when time tends to infinity and satisfies $0 \leq \rho(t)_i \leq rho_{max}$ (see Figure 1a). Here, we fixed $\rho_{min} = 0$ and $\rho_{max} = 1$ but both may vary between 0 and 1. δ_ρ is the lag time before $\rho(t)_1$ begins to decrease (or $\rho(t)_2$ increases) and θ_ρ is the parameter that inversely affects the decreasing rate.

Parameters of bimodal heterogeneity

From the parameters of the mixture distributions we calculated : S, the bimodal separation, such as:

$$S = \frac{(\mu_{max} - \mu_{min})}{2(\sigma_{max} + \sigma_{min})}.$$

For each mode i of the distribution S can be calculated relatively to any optimum mean with null standard deviation (here, 0.87(0), the theoretical optimum F_v/F_m value) as:

$$S_i = \frac{(0.87 - \mu_i)}{2(\sigma_i)}$$

and used to calculate the weighted contribution to bimodality of two distributions relatively to an optimum W_{max} as:

$$W_{max} = \frac{S_1 \times \rho_1 - S_2 \times \rho_2}{S_1 \times \rho_1}.$$

Simulations

Distributions were obtained using the *rtnorm* function of the **msm** package for random generation from a truncated Normal distribution $[0, 0.87]$ with mean equal to $\mu(t)$, standard deviation equal to $\sigma(t)$ and $\rho(t)$ the weight of each distribution (Figure 2). Simulations were performed for $n = 10000$ observations, the median number of pixels of the ChlF images which varied from 21 to 68000 pixels.
The dynamics of S and W_{max} were then calculated (Figure 3).

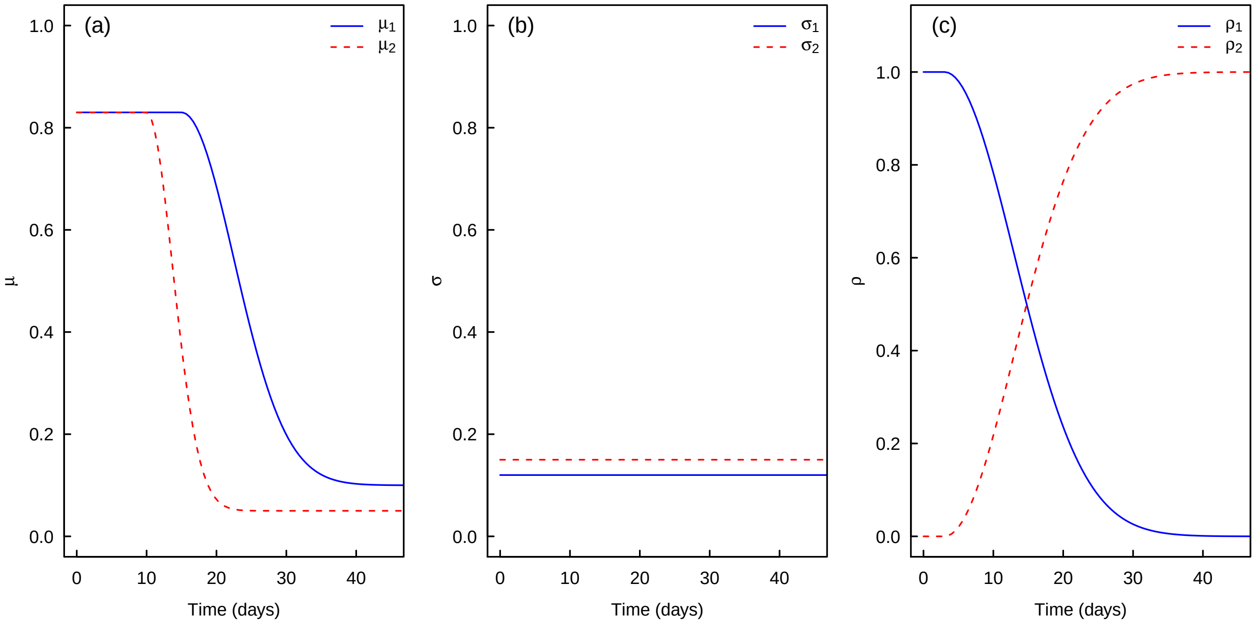

Figure 1: (a) Example of dynamics of the means (μ) of two Gaussian populations following the equations above with $\delta_\mu = (15, 10), \theta_\mu = (60, 30)$. (b) Constant standard deviations (σ) of two Gaussian populations following the equations above with $a = (0.07, 0.10)$, $b = 1$. (c) Example of dynamics of the weights of (ρ) of two gaussian populations following the equations above with $\delta_\rho = 3$ and $\theta_\rho = 80$. This set of parameters values approximate the values observed in the data by Bresson *et al.* (this paper) in response to severe water deficit for plants that did not survive the stress.

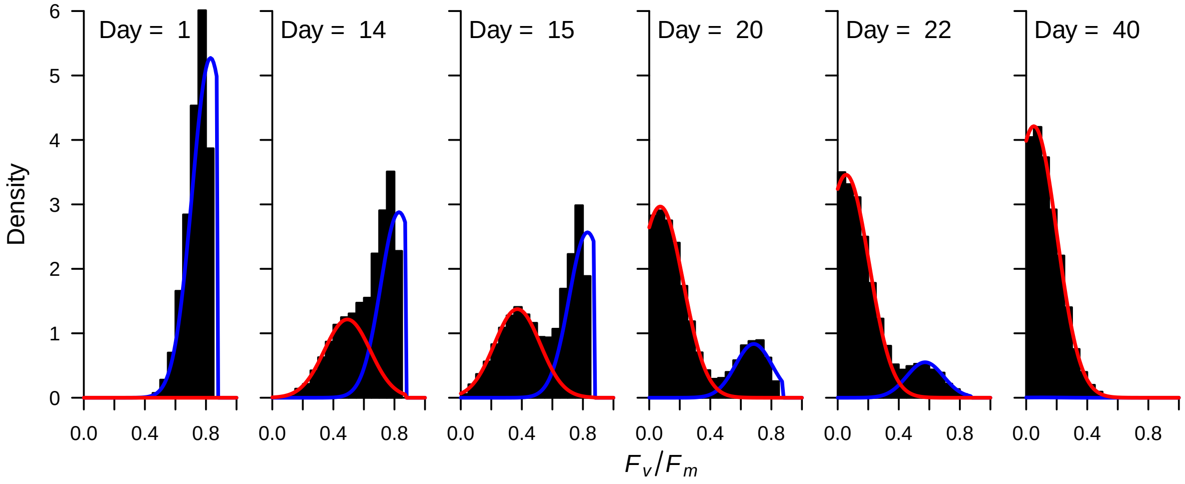

Figure 2: Example of distribution dynamics using the parameters of Figure 1.

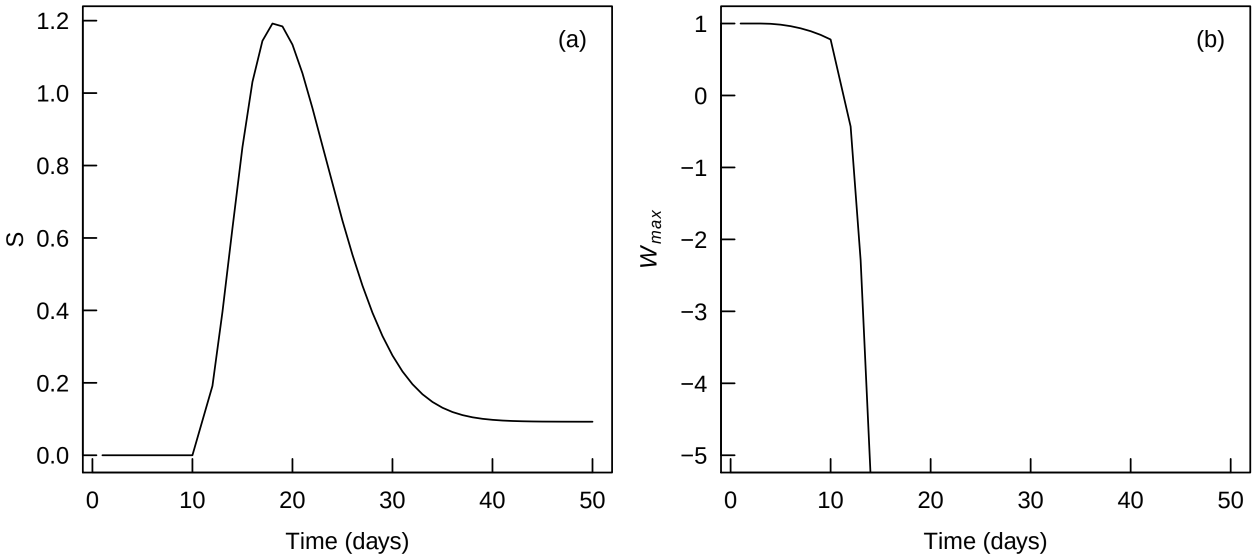

Figure 3: Dynamics of heterogeneity indices. Variation of S (a) and (b) W_{max} (b) given the parameters of Figure 1.

Experimental design of sensitivity analyses

The sensitivity of the parameters S and W_{max} to the variation of the mixture parameters was then investigated. We performed the sensitivity analysis on 45 sets of parameters chosen to mimic a wide range of potential photosynthetic stresses (*e.g.*, severe and moderate water stress, localized disease) as:

$$\begin{cases} \theta_{\mu 1} = \{80, 90, 100\}, & \delta_{\mu 1} = 20, & \mu_{max1} = 0.83, & \mu_{min1} = \{0.30, 0.50, 0.75\}. \\ \theta_{\mu 2} = \{20, 50, 90\}, & \delta_{\mu 2} = 10, & \mu_{max2} = 0.83, & \mu_{min2} = \{0.10, 0.15, 0.20\}. \\ a_1 = 0.08, & b_1 = 1, & & \\ a_2 = 0.12, & b_2 = 1, & & \\ \theta_{\rho 1} = \{100, 200, 300, 500, 1000\}, & \delta_{\rho 1} = 3, & \rho_{max1} = 1, & \mu_{min1} = 0. \end{cases}$$

where increasing values of the parameters for $\mu(t)_1$ ($n = 3$; green to blue in Figure 4a), $\mu(t)_2$ ($n = 3$; cyan to red in Figure 4a) and $\rho(t)$ ($n = 5$; orange to violet in Figure 4c) were combined, whereas $\sigma(t)_i$ were kept constant for clarity (blue for $\sigma(t)_2$ and red for $\sigma(t)_2$ in Figure 4b). θ are inversely related to the decreasing rates of the means μ_i and proportion ρ_1 of the healthiest pixels, *i.e.* the rate at which the stress is affecting the plant. δ represent the lag time before parameters are changing (*e.g.* the lag time before stress has measurable effects or, for a plant disease, the incubation time). The lag times $\delta_{\mu i}$ have been kept constant in the simulations presented in Figures 4-6, whereas $\delta_{\mu 1}$ varied at 20, 40 and 60 in simulations presented in Figures 7-8. Figure 4 gives the dynamics of the parameters of the distributions and Figures 5 and 6 give the dynamics of S and W_{max} following this experimental design.

Results and discussion

As expected, the patterns of variation of S and W_{max} were highly impacted by the dynamics of the mixture distributions (Figures 5-6). The level of photosynthetic heterogeneity (S) during stress is mainly controlled by the variation of $\mu(t)_1$ (blue to green curves, Figures 4a and 5b). However, the increase in S at the beginning of a stress is independent of the rate of decrease of $\mu(t)_1$ (inversely related to $\theta_{\mu 1}$) but depends on the rate of decrease of $\mu(t)_2$ ($\theta_{\mu 2}$, red, grey and blue curve, figures 4a and 5b). S reaches a maximum when the separation between the means is maximal and then it decreases to a plateau which occurence is dependant

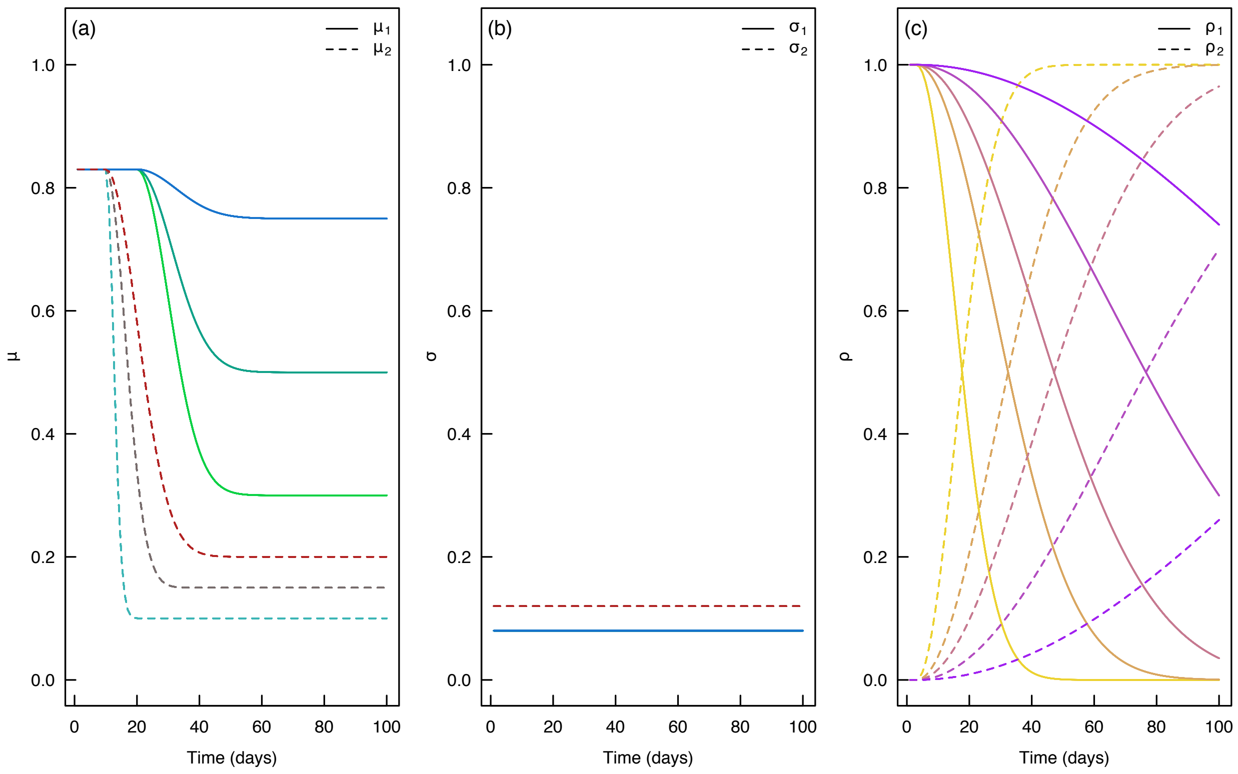

Figure 4: Combinations of bimodal parameters ($n = 45$) for sensitivity analyses. Different colours represent different combinations of parameters whereas same colour represents covarying parameters. (a) Means, $\mu(t)_1$ ($\theta_{\mu 1} = \{80, 90, 100\}, \delta_{\mu 1} = 20, \mu_{max1} = 0.83, \mu_{min1} = \{0.30, 0.50, 0.75\}$; green to blue), $\mu(t)_2$ ($\theta_{\mu 2} = \{20, 50, 90\}, \delta_{\mu 2} = 10, \mu_{max2} = 0.83, \mu_{min2} = \{0.10, 0.15, 0.20\}$; cyan to red). (b) Standard deviations, $\sigma(t)_1$ ($a_1 = 0.08, b_1 = 1$, blue), $\sigma(t)_2$ ($a_2 = 0.12, b_2 = 1$, red); kept constant for clarity. (c) $\rho(t)_1$ ($\theta_{\rho 1} = \{100, 200, 300, 500, 1000\}, \delta_{\rho 1} = 3, \rho_{max1} = 1, \mu_{min1} = 0$; orange to violet). θ are inversely related to the decreasing rates and δ represent the lag time. See text for details.

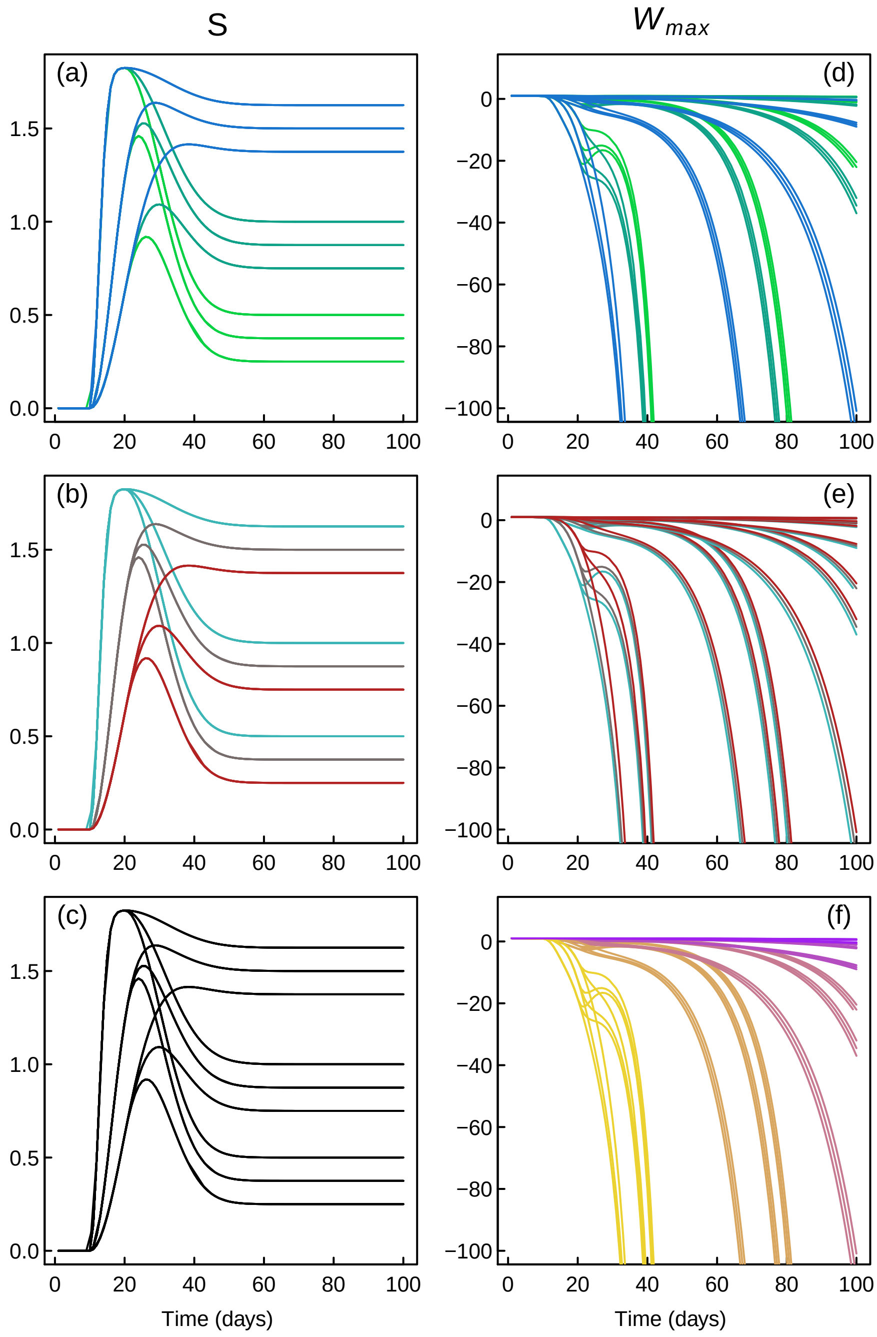

Figure 5: Variation of S (left) and W_{max} (right) given the combinations of bimodal parameters in Figure 4. Colours in panels vary following the variations of the parameters in Figure 4: each panel line represents $\mu(t)_1$ (a, d), $\mu(t)_2$ (b, e) and $\rho(t)$ (c, f). Note that $\sigma(t)_i$ are constant and that S is independent of ρ.

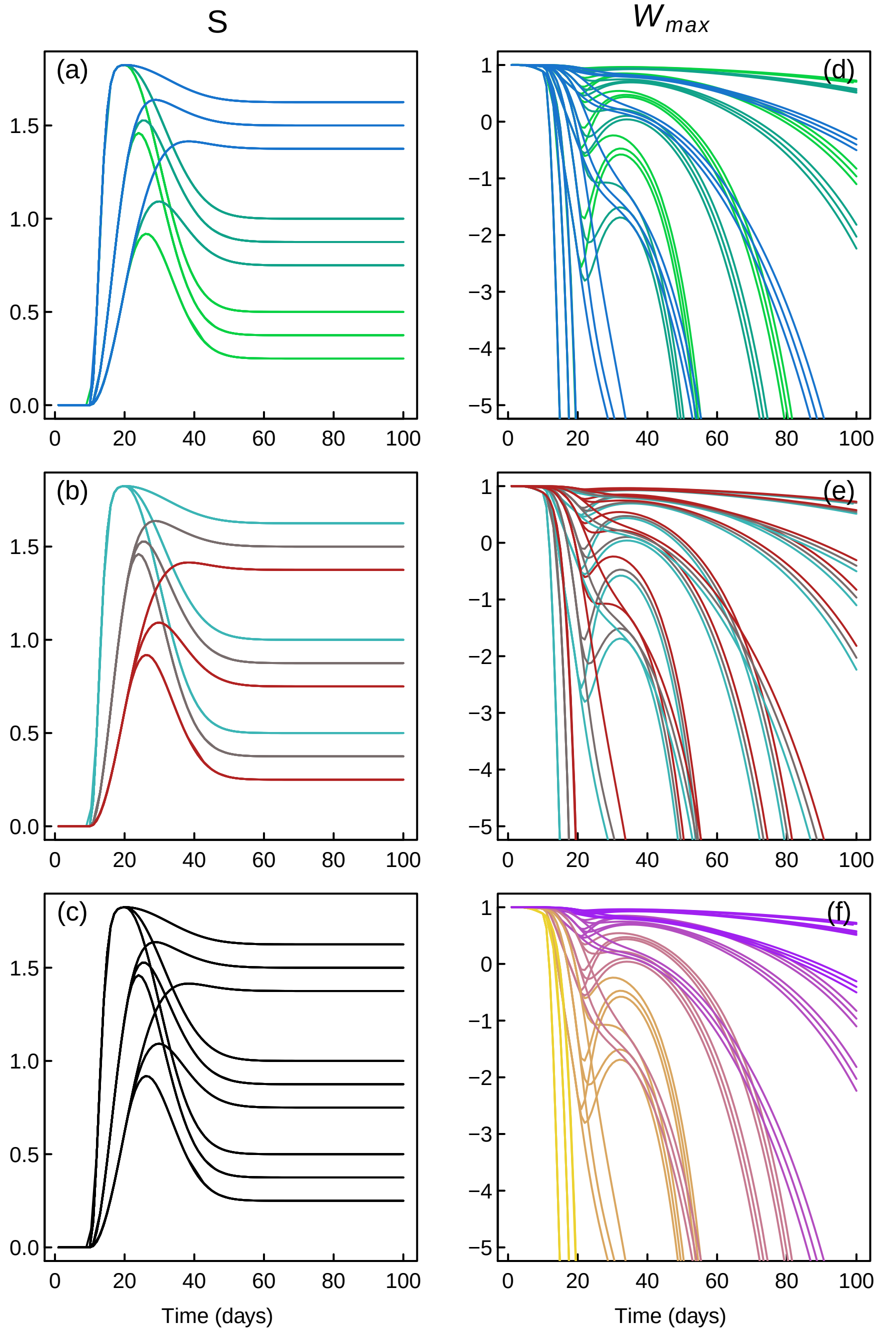

Figure 6: Variation of S (left) and W_{max} (right) given the combinations of bimodal parameters in Figure 4. Colours in panels vary following the variations of the parameters in Figure 4: each panel line represents $\mu(t)_1$ (a, d), $\mu(t)_2$ (b, e) and $\rho(t)$ (c, f). Note that $\sigma(t)_i$ are constant and that S is independent of ρ. Note also that the limits of y-axis for W_{max} has been set to [-5;1].

on the decreasing rates $\theta_{\mu i}$ of both means, on the lag times $\delta_{\mu i}$ (Figures 7-8) and value is dependent on the lower asymtote μ_{min} (Figures 5a,b and 8a,b). The lag time between the start of the stressing period and the appearence of the first effects on photosynthetic performance is an important parameter of the dynamics of stress tolerance. $\delta_{\mu 2}$ can illustrate the resilience of healthy parts (plant parts not impacted by the stress). If we take the example of a plant subjected to a disease, $\delta_{\mu 1}$ and $\delta_{\mu 2}$ participate in illustrating both the incubation period and the spread of the symptoms (*e.g.* localised vs. systemic).
Therefore, a sudden change, followed by a quick decrease, in S indicates a rapid effect of a factor or high sensitivity of the plant to a factor (*i.e.* low $\theta_{\mu i}$), whereas, a gradual increase, followed by a slow decrease, in S is indicative of a slight effect of a factor or of an effect spatially localised to small areas, and can also be indicative of high tolerance of the plant to a stress.
However, S does reflect the spread (in terms of area impacted) of the effects since this index does not take into account the size the impacted regions (*i.e.* ρ_i; Figure 5c). On the contrary, W_{max} explicitly takes into account the weights of the mixture distribution and therefore the size of the populations whose means are varying. W_{max} is also highly sensitive to the variations in the distribution parameters (Figure 5d-f). When the proportion of impacted pixels increases rapidly (*i.e.* low θ_ρ), indicative of a quick and generalised defect in the photosynthetic performance, W_{max} can decrease abruptely from 1 to $-\infty$ (Figure 5f). However, the dynamics of W_{max} also depend on the dynamics of μ_i. For instance, W_{max} increases when S is decreasing, which is particularly visible when the lag time $\delta_{\mu i}$ is changing (Figures 7-8). This simulation analysis suggests that the variation of W_{max} during stress (specifically, the rate of decrease and the time to reach negative values) can be a good indicator of stress tolerance in response to many environmental stresses.

Conclusions

The simplified simulation exercise used here was sufficient to reproduce what has been observed in this paper by Bresson *et al.* in plants grown under severe water deficit that did not survive the stress (Figures 1-3).
We argue that this simple simulation exercise is sufficient to reproduce the different behaviours of a plant or a leaf facing a change in its environment or its physiological status in terms of sensitivity, tolerance and dynamics. Specifically, the parameters of the equations used to simulate the dynamics of mixture distributions can be used as indicators of the tolerance/sensitivity of a plant to any factor or combination of factors. For instance, some factors can have spatially localised determinisms (*e.g.* pathogens) with more or less random patterns while others can be more diffuse. Moreover, some factors can act suddently or be more diffuse through time (*e.g.* suddent changes in temperature vs. moderate but continuous water deficit).
Future developments, in addition to the accumulation of experimental evidences, will improve the simulation process to explicitly include spatial dynamics, functional hypotheses of variation of the parameters, and stress recovering, and could allow the prediction of plant response to environmental factors.

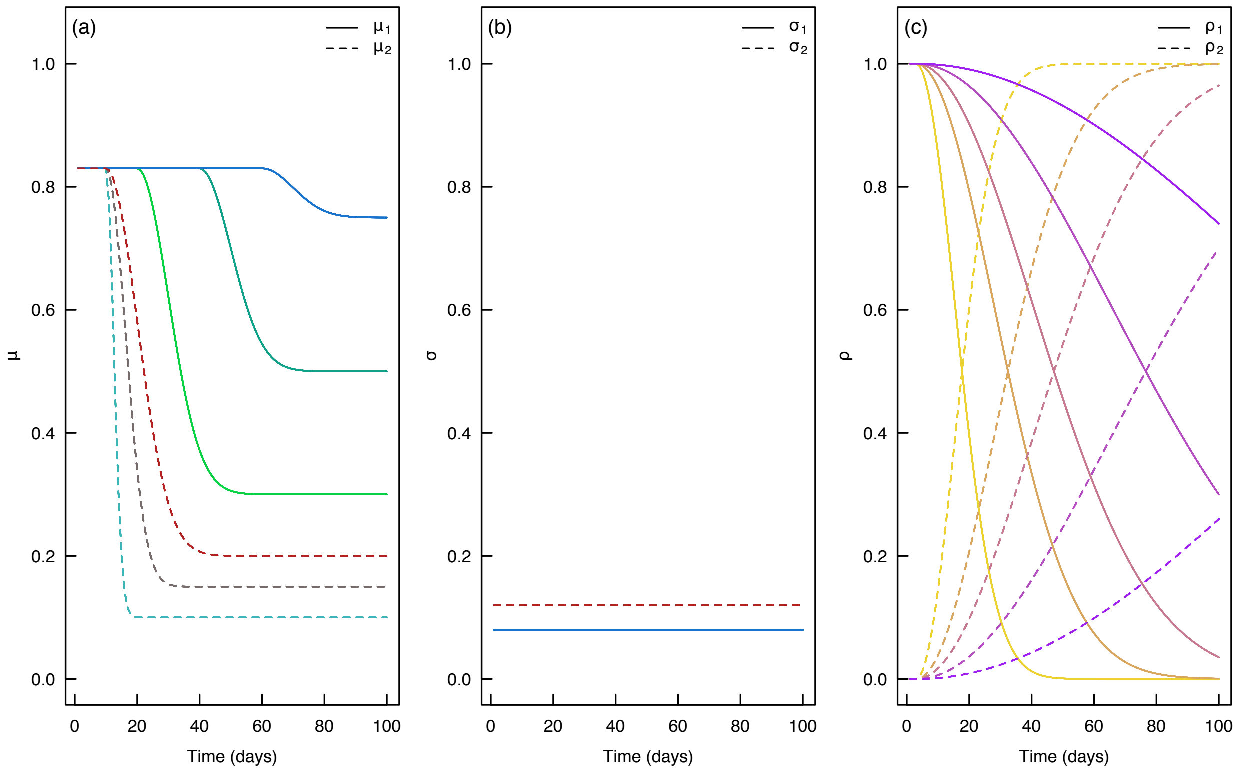

Figure 7: Combinations of bimodal parameters ($n = 45$) for sensitivity analyses showing the effects of changes in the lag time $\delta_{\mu 1}$. Different colours represent different combinations of parameters whereas same colour represents covarying parameters. (a) Means, $\mu(t)_1$ ($\theta_{\mu 1} = \{80, 80, 80\}, \delta_{\mu 1} = 20, 40, 60, \mu_{max1} = 0.83, \mu_{min1} = \{0.30, 0.50, 0.75\}$; green to blue), $\mu(t)_2$ ($\theta_{\mu 2} = \{20, 50, 90\}, \delta_{\mu 2} = 10, \mu_{max2} = 0.83, \mu_{min2} = \{0.10, 0.15, 0.20\}$; cyan to red). (b) Standard deviations, $\sigma(t)_1$ ($a_1 = 0.08, b_1 = 1$, blue), $\sigma(t)_2$ ($a_2 = 0.12, b_2 = 1$, red); kept constant for clarity. (c) $\rho(t)_1$ ($\theta_{\rho 1} = \{100, 200, 300, 500, 1000\}, \delta_{\rho 1} = 3, \rho_{max1} = 1, \mu_{min1} = 0$; orange to violet). θ are inversely related to the decreasing rates and δ represent the lag time. See text for details.

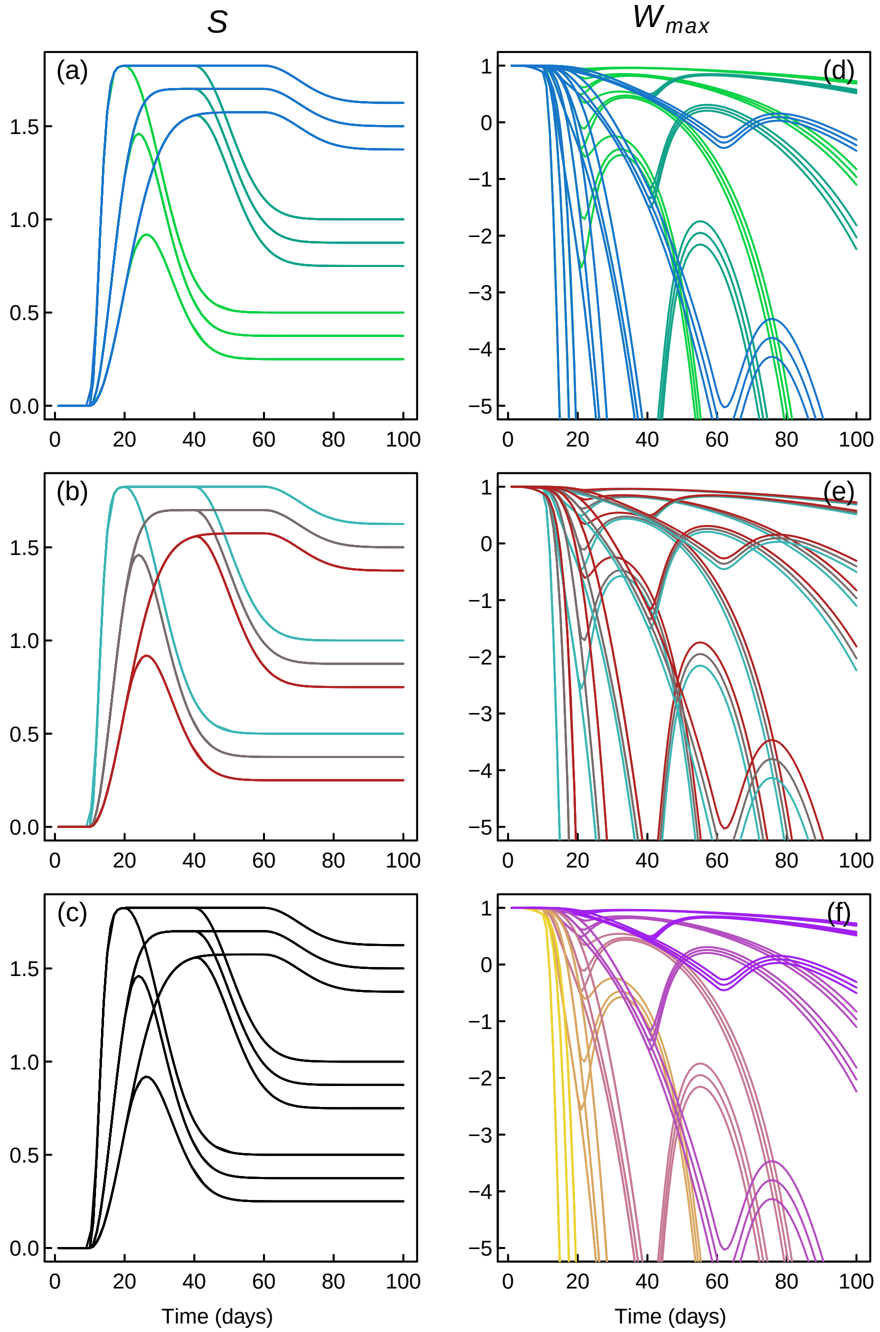

Figure 8: Effects of changes in the lag time $\delta_{\mu 1}$ on the variation of S (left) and W_{max} (right) given the combinations of bimodal parameters in Figure 7.
