# Supplementary material for: Quantifying spatial heterogeneity of chlorophyll fluorescence during plant growth and in response to water stress
Source: Plant Methods. 2015 Mar 26;11:23. doi: 10.1186/s13007-015-0067-5 (PMC4394423; doi:10.1186/s13007-015-0067-5)
Supplement: Additional file 6: Table S1. — Soil chemical properties of the compost (Neuhaus N2), soil and two mixtures of both. Mixture 1 was sampled before experimentation and mixture 2 was sampled after experimentations. nd: not determined. Soil analysis was performed by ALFA Agricultural Service and Research Building, Soil Testing Laboratory of Auburn University. [file 13007_2015_67_MOESM6_ESM.pdf]

	Abbreviation	Methods	Units	Substrats Compost N2	Soil	Mixture 1	Mixture 2
**Calcium**	Ca	Dry Ash analysed by inductively coupled plasma (ICP)	% in soil	1.95	17.21	17.11	12.69
**Potassium**	K		% in soil	0.16	0.15	0.20	0.22
**Magnesium**	Mg		% in soil	0.25	0.35	0.42	0.34
**Phophorus**	P		% in soil	0.08	0.067	0.093	0.063
**Aluminum**	Al		ppm	997	7486	6854	7934
**Arsenic**	As		ppm	< 0.1	< 0.1	< 0.1	< 0.1
**Boron**	B		ppm	11	22	26	22
**Barium**	Ba		ppm	183	732	746	770
**Cadnium**	Cd		ppm	< 0.1	< 0.1	< 0.1	< 0.1
**Chromium**	Cr		ppm	16	18	28	29
**Copper**	Cu		ppm	14	33	21	23
**Iron**	Fe		ppm	835	5123	6353	7070
**Manganese**	Mn		ppm	81	361	466	467
**Molybdenum**	Mo		ppm	< 0.1	< 0.1	< 0.1	< 0.1
**Sodium**	Na		ppm	2029	766	1483	1480
**Nickel**	Ni		ppm	78	518	551	760
**Lead**	Pb		ppm	< 0.1	< 0.1	< 0.1	< 0.1
**Zinc**	Zn		ppm	31	46	45	43
**Ammonium-Nitrogen**	NH4-N	Saturated paste extraction analyzed	ppm	0.2	0.1	0.2	0.3
**Nitrate-Nitrogen**	NO3-N		ppm	114	56	216	428
**Nitrogen**	N	Dry combustion	% in soil	1.10	0.11	0.18	0.24
**Carbon**	C		% in soil	40.36	7.27	10.48	9.75
**Electrical Conductivity**	EC	Solubridge	mmhos/cm	2.7	1.2	2.5	5.1
**Soluble Salts**	SS		ppm	1910	868	1736	3559
